# Reducing the cost and assessing the performance of a novel adult mass-rearing cage for the dengue, chikungunya, yellow fever and Zika vector, *Aedes aegypti* (Linnaeus)

**DOI:** 10.1371/journal.pntd.0007775

**Published:** 2019-09-25

**Authors:** Hamidou Maïga, Wadaka Mamai, Nanwintoum Séverin Bimbilé Somda, Anna Konczal, Thomas Wallner, Gustavo Salvador Herranz, Rafael Argiles Herrero, Hanano Yamada, Jeremy Bouyer

**Affiliations:** 1 Insect Pest Control Laboratory, Joint FAO/IAEA Division of Nuclear Techniques in Food and Agriculture, International Atomic Energy Agency, Vienna, Austria; 2 Institut de Recherche en Sciences de la Santé/Direction Régionale de l’Ouest, Bobo‑Dioulasso, Burkina Faso; 3 Institut de Recherche Agricole pour le Développement, Yaoundé, Cameroun; 4 Technical School of Design, Architecture and Engineering, University CEU Cardenal Herrera, Valencia, Spain; USDA-ARS Center for Medical Agricultural and Veterinary Entomology, UNITED STATES

## Abstract

**Introduction:**

The widespread emergence of resistance to insecticides used to control adult *Aedes* mosquitoes has made traditional control strategies inadequate for the reduction of various vector populations. Therefore, complementary vector control methods, such as the Sterile Insect Technique, are needed to enhance existing efforts. The technique relies on the rearing and release of large numbers of sterile males, and the development of efficient and standardized mass-rearing procedures and tools is essential for its application against medically important mosquitoes.

**Methods:**

In the effort to reduce the cost of the rearing process, a prototype low-cost plexiglass mass-rearing cage has been developed and tested for egg production and egg hatch rate in comparison to the current Food and Agriculture Organization/International Atomic Energy Agency (FAO/IAEA) stainless-steel cage. Additionally, an adult-index was validated and used as a proxy to estimate the mosquito survival rates by counting the number of male and female mosquitoes that were resting within each of the 6 squares at a given point of time each day in the cage.

**Results:**

The study has shown that the prototype mass-rearing cage is cheap and is as efficient as the FAO/IAEA stainless-steel cage in terms of egg production, with even better overall egg hatch rate. The mean numbers of eggs per cage, after seven cycles of blood feeding and egg collection, were 969,789 ± 138,101 and 779,970 ± 123,042, corresponding to 81 ± 11 and 65 ± 10 eggs per female over her lifespan, in the prototype and the stainless-steel-mass-rearing cages, respectively. The longevity of adult male and female mosquitoes was not affected by cage type and, the adult-index could be considered as an appropriate proxy for survival. Moreover, the mass-rearing cage prototype is easy to handle and transport and improves economic and logistic efficiency.

**Conclusion:**

The low-cost mass-rearing prototype cage can be recommended to produce *Ae*. *aegypti* in the context of rear and release techniques. The proposed adult-index can be used as a quick proxy of mosquito survival rates in mass-rearing settings.

## Author summary

Dengue, among other arboviral infections, is a neglected disease and a major health issue that is re-emerging in tropical countries due to the poor efficacy of conventional vector control methods. Therefore, there is a growing need for more sustainable techniques to control *Aedes* mosquito species, while reducing the dependence on insecticides. The sterile insect technique, which relies on the mass-production of sterile males, can be used as part of area- wide integrated pest management (AW-IPM) programmes to reduce the vector population below the disease transmission threshold. Therefore, innovations in mosquito mass-rearing techniques including the development of low-cost adult holding cages are essential in the quest to promote an economical and logistically efficient mass -rearing system for the vectors of dengue, chikungunya, yellow fever and Zika diseases.

## Introduction

*Aedes aegypti* (Linnaeus) is a highly invasive, medically important mosquito species which has received a considerable increase in attention after being linked to the Zika outbreak in Brazil in 2015 [1]. Together with *Aedes albopictus* (Skuse), the species also transmits several arboviral diseases including dengue, chikungunya and yellow fever [2]. Dengue viruses alone are estimated to infect 390 million people per year, including 96 million cases with clinical manifestations [3]. The heavy reliance on insecticides to control adult *Aedes* mosquitoes, especially during disease outbreaks, has led to the emergence of widespread resistance to the limited chemical classes that are currently available, and complementary vector control methods to enhance existing efforts are needed [4]. Amongst those being advocated is the sterile insect technique (SIT), a species-specific and environmentally-friendly pest population control method. According to the International Standards for Phytosanitary Measures No. 5 Glossary of phytosanitary terms, the SIT is a “Method of pest control using area-wide inundative releases of sterile insects to reduce reproduction in a field population of the same species”. The potential of the SIT for mosquito suppression has been demonstrated in a feasibility study in Italy [5], but successful implementation of the technique will rely on maintaining continuous production and repeated release of over-flooding numbers of sterile males [6] that can outcompete their wild counterparts within the target area [7]. To meet these requirements, novel methods and materials for the mass-rearing of mosquitoes are needed [8].

Optimization of mass-rearing conditions for both immature stages (for pupal production) and adult egg production requires a balance between accommodating the biological needs of the species, and achieving high production rates and economic efficiency [9]. For instance, the quality of larval diet [10–12] impacts pupal size and thus female adult size, which in turn determines bloodmeal intake and egg production [13,14]. The fine-tuning of the rearing cycle also requires adult cages to support as close-to-natural as possible behaviour of the mosquitoes. The ideal cage would also be practical in terms of handling, maintenance and space requirements, whilst keeping costs low. Cage design and construction requires careful adjustment of its dimensions in relation to the number of mosquitoes it should hold, providing suitable conditions for mating, feeding, and good survivorship [8,15]. This factor, which we call the Density-Resting Surface per mosquito (adult/cm^2^), or DRS, is obtained by dividing the number of adult mosquitoes by the vertical resting surface area on the four sides of a cage [15].

To date, only limited mosquito-rearing methods are available [9,15–19]. Adult mass-rearing cages made of stainless-steel, resistant to corrosion and allowing high temperature cleaning, have been developed for *Ae*. *albopictus* [9,19] but these have not been fully validated for *Ae*. *aegypti*. Preliminary results for egg production in *Ae*. *aegypti* under routine mass-rearing conditions have shown the capacity of the Food and Agriculture Organization/International Atomic Energy Agency (FAO/IAEA) stainless-steel cage [9] in supporting egg production. However, the stainless-steel cage faces a high production cost (~€2,300/unit). Another serious drawback of the stainless-steel cage is the significant loss of viable material resulting from eggs hatching prematurely within the cage after the first egg collection. In response to these major issues, a mass-rearing cage prototype has been developed to both reduce the purchase cost and to improve egg yield and adult survival. The final goal is to develop equipment for a mosquito mass-rearing facility, such as those found in established fruit-fly production facilities [7]. This study presents a novel mass-rearing cage and aims to test the cage in terms of egg production and hatch rate in comparison with the FAO/IAEA stainless-steel cage. Additionally, an adult index was validated and used as a quick and reliable proxy to estimate survival rates.

## Methods

### Ethics statement

Studies on mosquito species do not require a specific permit according to the document 2010/63/EU of the European Parliament and the Council on the protection of animals used for scientific purposes. All mosquito strains used in the present study were maintained in the biosecure insectaries of the Joint FAO/IAEA Insect Pest Control Laboratory (IPCL), Seibersdorf, Austria. All the experiments were performed based on standard operating procedures in the IPCL (FAO/IAEA, 2018). The blood used for routine blood-feeding of mosquitoes was collected in Vienna, Austria during routine slaughtering of pigs or cows in a nationally authorized abattoir (Rupert Seethaler, Himberg bei Wien) at the highest possible standards strictly following EU laws and regulations.

### Mosquito strain and rearing conditions

The *Ae*. *aegypti* strain used in this study originated from field collections in Juazeiro (Bahia), Brazil and were transferred to the ICPL from the insectary of Biofabrica Moscamed, Juazeiro, Brazil in 2016. The strain is maintained following the “Guidelines for Routine Colony Maintenance of *Aedes* mosquitoes” [20].

Immature stages were reared under controlled temperature, humidity and lighting conditions (T = 28± 2°C, 80± 10 RH%, and 12:12 h light:dark, including 1h dawn and 1h dusk) whereas adults were maintained in a separate room at 26± 2°C, 60± 10 RH%, 12:12h light:dark, including 1h dawn and 1h dusk.

### Description of the mass-rearing cage prototype

#### Cage structure

**Fig 1:**
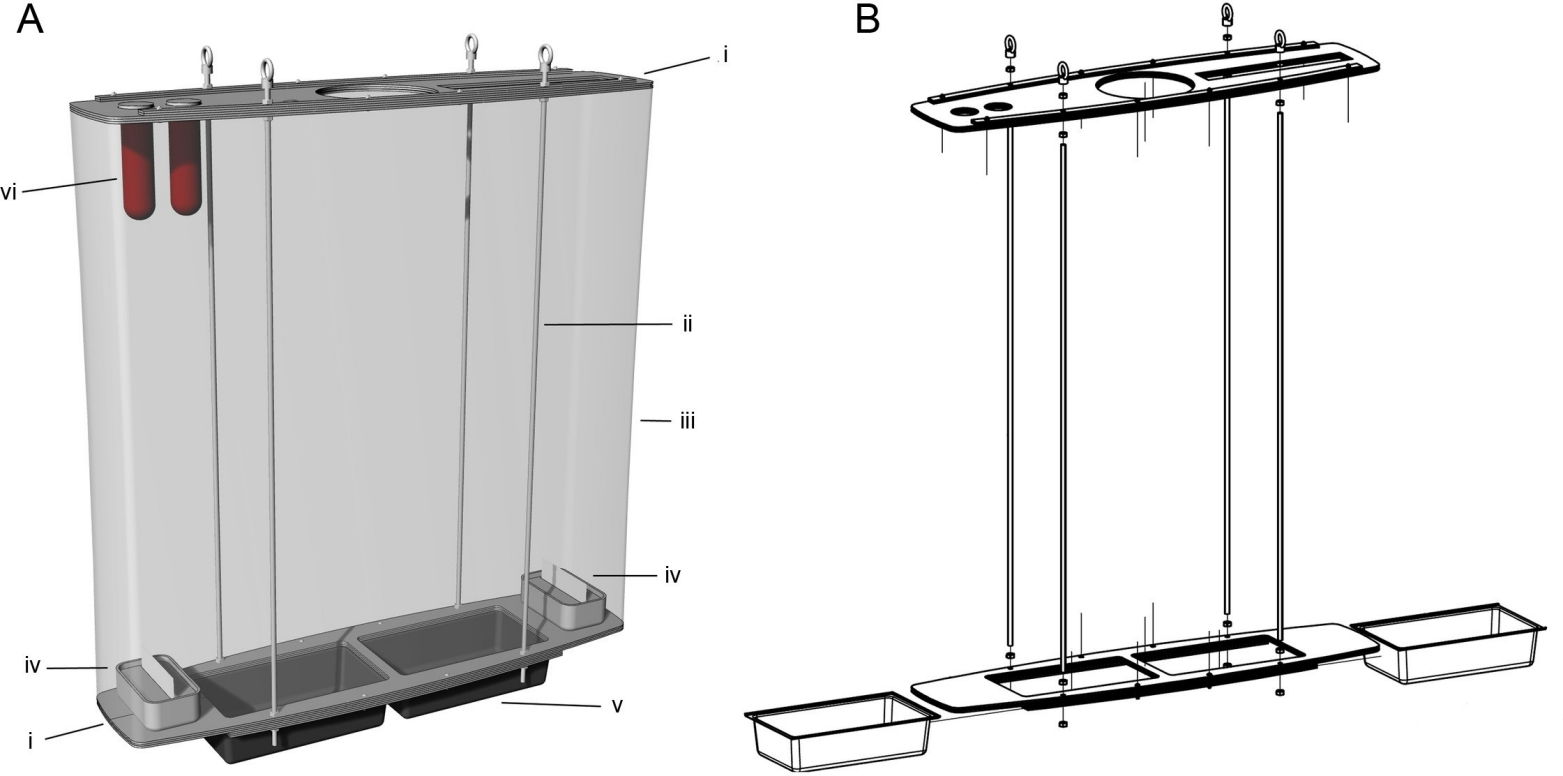
**The 3D design of the mass-rearing cage prototype (A) and structure (B).** Plexiglass laser cut plates (i), four metal rods (ii), mesh netting (iii), sugar feeding container (iv), two containers for pupae and egg collection (v); mesh socks blood feeders (vi). More details are available as supporting information files (S1–S15 Figs).

The mass-rearing cage prototype (Fig 1, Table 1) is made of Poly(methyl methacrylate) (PMMA / plexiglass) laser cut plates (Fig 1A (i)). The cage has a volume of 162 litres, with overall dimension of 900 (L) x 900 (H) x 200 (W) mm with four threaded rods (Fig 1A (ii)) to allow adjustment of the cage height, two containers for pupal loading and egg collection (Fig 1A (iv)) and mesh netting (Fig 1A (iii)) that is held along the top and bottom plexiglass plates with a thin rubber band to ease replacement when needed. The cage weighs about 5 kg and can stand on a table or be hung vertically from the ceiling using chains. Multiple cages could be hung on an overhead track, allowing them to be moved around for ease of access. When not in use, cages can be disassembled for storage.

**Table 1:** Cost of material for the mass-rearing cage prototype.

Item	description	unit	estimated cost Per Unit (€)
**Plexiglass upper plate**	as per technical drawings	1	90
**Plexiglass bottom plate**	as per technical drawings	1	90
**Threaded rods M8**	Stainless-steel. GB/T 15389–1994 M8 x 1000	4	6
**Hexagonal nut M8**	Stainless-steel. ISO 4032—M8	16	0.3
**Eyebolt nut M8**	JIS B 1169—M 8	4	3
**Fabric**		1	10
**Egg collection container**	ABS 1mm	2	2
**Total cage cost**			234.8

The cost was estimated according to local cost of production in Austria.

#### Pupal loading

The bottom of the cage has two rectangular holes (L × W = 284 × 158 mm) to which two black containers (L × W × H = 306.82 × 185 × 76 mm) (Fig 1A (v)) holding pupae can be attached for adult emergence. Each container has the capacity to hold up to 14,000 pupae, at a density of 25 pupae/cm^2^). Double plexiglass fixtures at the bottom surface of the cage allow for sliding of the containers for removal or replacement.

#### Sugar feeder

Two fixed internal containers (L × W × H = 150 × 90 × 50 mm) made of a transparent plastic (600 mL maximum capacity) are used as sugar feeders (Fig 1A (iv)). Each container is sealed onto each end of the bottom plexiglass and is filled with 500 mL of 10% (w/v) sugar solution (100g of sucrose dissolved in 1,000mL of reverse osmosis purified water) to last for the duration of the rearing cycle of the cage. A slot (L × W = 135 × 2 mm) on top of each sugar container allows insertion of a cellulose sponge cloth (SKILCRAFT, MR 580, USA) which remains saturated with 10% sugar solution for mosquito feeding.

#### Blood feeder

The plexiglass plate at the top of the cage has two round holes (50 mm in diameter) to serve as blood feeding ports (Fig 1A (vi)). Two 170 mm long mesh socks are each hung vertically from these ports and allow the insertion of blood-filled sausage casings for blood feeding.

#### Egg collection system

The removable pupae containers (L × W × H = 306.82 × 185 × 76 mm) described in the “pupal loading” section serve a dual purpose and are also used as oviposition sites (Fig 1A (v)). Eggs are collected on filter papers lined along the long sides inside the container which is filled with 1L of reverse osmosis purified water. The egg papers are held by a 10 mm thick plastic plate lining the bottom of the container. The design of the plastic plate allows the insertion of up to 5 egg papers in each container (10 papers in total per cage) if needed. To collect eggs, the containers are slid onto the bottom of the cages. To replace the container with a new one, the existing one can be pushed along the sliders and out of the cage by a new container holding new papers and fresh water. While sliding, each container can be covered to avoid escapees with a 4 mm plastic plate which fits onto the opening. Oviposition containers are gently agitated to induce resting mosquitoes to fly out before being removed from the cage.

#### Manufacture

The cage is designed following the Open Source Hardware (OSHW) Statement of Principles 1.0. All of the elements of the cage are made from common materials that are readily available around the world thus can be locally sourced (Table 1). The plexiglass plates can be manufactured in any part of the world using a laser cutter or sourced from online prototyping companies. The technical drawings of the mass-rearing cage prototype are freely available as supporting information files (S1–S15 Figs) and on the website of the FAO/IAEA joint Division under a Creative Commons 4.0 Attribution International license.

### Larval mass-rearing and pupae production for experiments

All pupae were produced with the FAO/IAEA free-standing larval mass-rearing trays (L × W × H = 100 × 60 × 3 cm, Glimberger Kunststoffe Ges.m.b.H., Austria) [21]. Enough eggs to produce 18,000 larvae (L1) were added to 5L of reverse osmosis purified water per tray (5,000 cm^2^ inner surface of the tray) to give a rearing density of 3.6 larvae/cm^2^. To estimate the number of eggs required, 2-week-old eggs were brushed off egg collection papers and 3 samples of 100–150 eggs were hatched overnight using a 50mL centrifuge tube (VWR, UK) filled with 40mL of boiled and cooled reverse osmosis purified water with 2mL of 4% larval FAO/IAEA diet [22]. On the following day (about 20 hours later), the egg hatch rate of 100 eggs from each sample was verified under a stereomicroscope, and used to determine how many eggs would be needed to obtain 18, 000L1/tray. The required egg numbers were estimated using an equation (Weight (mg) = (0.0088 × Number of counted eggs) - 0.3324) described by Zheng et al. [23]. The egg batches to produce 18, 000 L1 for each tray were hatched separately. To hatch eggs, jam jars (IKEA of Sweden AB SE-343 81 Almhult, Germany) filled with 700mL of boiled and subsequently cooled (deoxygenated) reverse osmosis purified water stored at laboratory temperature were opened to add the eggs, before the jars were quickly closed again to avoid re-oxygenation. A volume of 10 mL (0.022 mg/larva) of 4% larval FAO/IAEA diet [22] was added to jars to synchronize egg hatching and improve larval development. All jars were kept for 20 hours before their contents were sieved and transferred to mass-rearing trays previously filled with 5L of osmosis water and covered (1 day before being seeded with larvae). A volume of 50 mL of larval food was added to each tray on day 1, 100 mL on day 2, 200 mL on days 3 and 4, 150 mL on day 5 and 50 mL on each additional day until day 9, corresponding to 0.11, 0.22, 0.44, 0.33 and 0.11 mg of ingredients per larva per day respectively [22].

Trays were individually tilted on day 6 after larval seeding, and larvae, male, and female pupae were separated using mechanical sexing tools [24] between 9am and 1pm. Larvae were returned to the rearing trays refilled with the retained rearing water. Batches of 500 pupae of each sex were counted manually and their volume estimated using a small cylindrical plastic tube (15 mL centrifuge tube, VWR, UK) covered with mesh on one side to hold pupae. The level of 500 pupae was daily marked on the tube and thus allowed estimation of the number of pupae.

### Assessing the adult-index as a proxy of adult survival rates

An adult-index was developed by drawing three 5.7 × 5.7 cm squares in the middle of the netted sides of a 30 × 30 × 30 cm Bugdorm cage (BugDorm-1 Insect Rearing Cage, Taiwan) using a fine marker. Each cage was loaded with approximately 3,000 *Ae*. *aegypti* (Brazil strain) mosquitoes (female:male ratio of 3:1 with a DRS of 0.8 mosquito/cm^2^). Mosquitoes were offered a 10% sugar solution using a 100mL urine cup holding a cellulose sponge cloth (SKILCRAFT, MR 580, USA). No blood feeding was performed during this experiment. A count of the number of male and female mosquitoes that were resting within each of the squares at a given point of time each day was used to estimate the density of adults in the cage, a measure named the ‘adult-index’. Dead mosquitoes were removed from the cages using a mouth aspirator and were counted and recorded according to sex for each of the 5 cages for a period of 4 weeks (except during the weekends). The adult mosquitoes that survived the 4 week-period were also removed, counted and sex-separated allowing the initial number of mosquitoes per cage at the beginning of the experiment to be calculated.

### Design of experiment to assess the mass-rearing cage prototype against the FAO/IAEA stainless-steel cage

Three and 4 replicates were performed for the stainless-steel cage and the mass-rearing cage prototype, respectively. Each cage was loaded with around 13,333 female and 4,444 male pupae (female:male ratio of 3:1 with a DRS of 0.8 mosquito/cm^2^) equally distributed over 4 consecutive days (Table 2) allowing the total final stocking of the cage with 12,000 and 4,000 adult females and males, respectively. This figure is reached using a pre-determined estimate 90% of adult emergence and survival rate until the first blood feeding. For the mass-rearing cage prototype, pupae were loaded into the black containers before these were fixed onto the bottom of the cage. Subsequent loading was made possible by sliding the containers backwards and adding pupae. Pupae were split equally into the two containers to avoid crowding and excess mortality. Pupae were introduced into the stainless-steel cage through an inlet valve to load the bottom part of the cage as previously described by Balestrino et al. [9].

**Table 2:** Design of experimental rearing procedures.

Day	1	2	3	4	8	10	11	12	15	18	22	25	29	32
**Pupal loading**	PL1	PL2	PL3	PL4										
**Blood feeding**					BF1		BF2		BF3	BF4	BF5	BF6	BF7	
**Egg papers addition**						EP1		EP2	EP3	EP4	EP5	EP6	EP7	
**Egg paper removal**								EC1	EC2	EC3	EC4	EC5	EC6	EC7
**Week of egg collection**	** **	** **	** **	** **	** **	** **	** **	**1**			**2**		**3**	

The schedule includes pupal loading (PL), blood feeding (BF), egg paper addition (EP) and egg paper removal (called egg collection) (EC) starting with the first day of loading pupae, up to the 7^th^ egg collection, covering a total active period of 32 days. Only days where tasks were performed (PL (1–4), BF (8;11;15;18;22;25;29), EP (10;12; 15;18;22;25;29) or EC (12; 15;18;22;25;29;32)) are shown.

Female mosquitoes were offered bloodmeals using two sausage casings (Grade Specification: 3)26 NC, EDICAS co ltd) each filled with 150 mL of fresh porcine blood [9]. The blood sausages were heated in a water bath (42°C) for 10 min before inserting them into the 17 cm long mesh socks hanging from the top of the cage. The females were fed for 30 min (blood temperature ranged between 25 and 37° C during feeding) before the same process was repeated to allow females to feed on warm blood for a total of 2 hrs in each cage type. Blood feeding was performed 7 times for each cage to give an active period of 32 days (Table 2).

Eggs were collected from each cage using seed germination papers (Grade 6, Size: 580 × 580mm, Weight: 145 g/m^2^, Sartorius Stedium Biotech). For the mass-rearing cage prototype, two egg papers (L × H = 270 × 60 mm) were inserted inside each container (4 egg papers in total) filled with 1L reverse osmosis purified water. To allow access to the same egging surface, 4 egg papers of L × H = 270 × 110 mm size each were inserted at the bottom of the FAO/IAEA stainless-steel cage, which was filled with 2L osmosis water, through four 400 mm-long and 3 mm-wide slots, two on each of the long sides as described by Balestrino et al. [9]. The first egg papers were added on the third day following blood meals and were removed three days later (Table 2). Further tasks were performed following the schedule as described in Table 2. When egg papers were collected from each cage, the remaining water was drained through stacked 300 and 50μm sieves (Retsch, Haan, Germany) to collect floating eggs, which were then dried on coffee filter papers (Melitta 1×4 Original FSC C095206) [25]. The eggs were allowed to mature and dry for 14 days [20] before the eggs were brushed off the papers and sieved through a 300 μm sieve (Retsch, Haan, Germany) to remove any debris. Seven egg batches were collected during the entire active period of each cage type. The mean number of eggs per female was estimated from the initial female pupae count and using the equation (Weight (mg) = (0.0088 × Number of counted eggs) - 0.3324) to estimate the number of eggs [23].

Egg hatch rate according to cage type and egg origin (either collected on papers or floating in water) was assessed following the protocol described above (**Larval mass-rearing and pupae production for experiments**).

**Fig 2:**
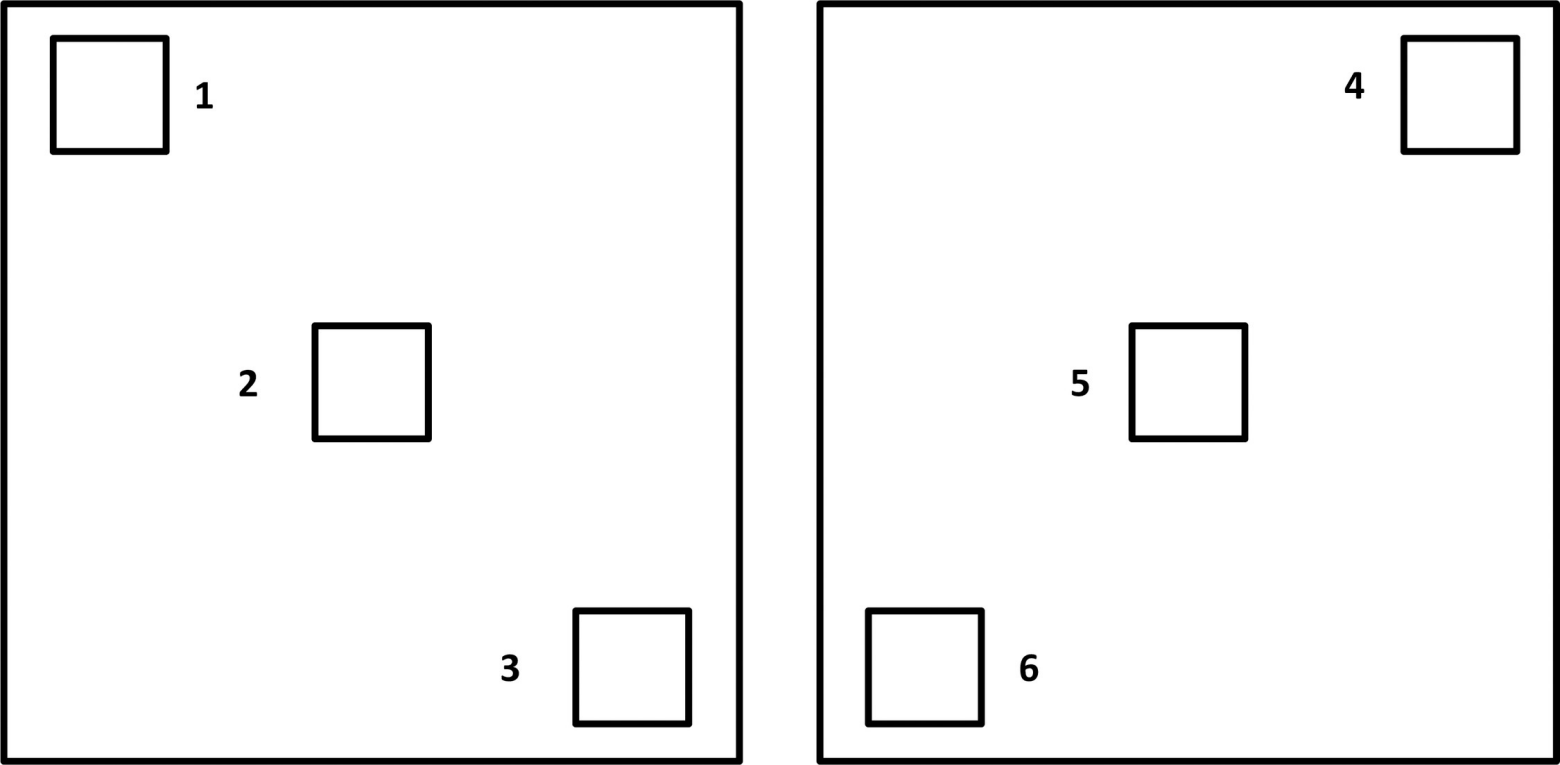
Drawings of six 10 × 10 cm squares for the cage survival rate monitoring. Three squares were drawn on each cage side on the cage netting for the adult-index check. Numbers indicate the location of the squares on the mass-rearing cage.

To estimate adult mosquito daily survival rates in the mass-rearing cage prototype in comparison to the FAO/IAEA stainless-steel cage, the adult-index data were used. Six 10 × 10 cm squares (three on each cage side) were drawn onto the netted sides of the cages using a fine marker (Fig 2). A daily cage density (termed adult-index) check was performed as described above at the same time every day for 4 weeks, except weekends. Counting adults in a given square took 10 to 45 seconds depending on the number of individuals, (longer at the beginning of the cage cycle).

### Data analysis

All statistical analyses were performed using R version 3.5. 2 [26].

The correlation between adult-index and survival rates was estimated. The number of daily live mosquitoes in the Bugdorm cages was analyzed using a Gaussian linear mixed-effect model (lme4 package) with the number of live mosquitoes defined as the dependent variable the adult-index (number of mosquitoes counted by square), square location (as a qualitative factor) and their interaction as fixed effects and replicate as a random effect. The best model was selected and the correlation between the number of adult mosquitoes that survived daily in the cage and the adult-index was estimated. The number of adult mosquitoes of a given day equals to the initial number of caged adults minus the number of dead adult mosquitoes until that day. A Pearson's product-moment correlation-test was used to analyze the correlation between the two values (number of live mosquitoes and the fitted model) for both female and male adult mosquitoes.

To correlate the daily mortality rates estimated from counting dead mosquitoes daily and the daily mortality rates from the counts in the squares, a mixed effect Gaussian linear model was used, with the mortality estimated in a given square, the square location and the sex as fixed effects and replicates as a random effect.

The egg production was analyzed using a Gaussian linear mixed-effect model (*lme*4 package) [27] with egg numbers per female defined as the dependent variable, cage type and week of egg collection (as a qualitative factor) and their interaction as fixed effects and replicates as a random effect. A generalized binomial linear mixed-effects model fit by maximum likelihood (Laplace Approximation) with logit link was performed with the proportion of floating eggs defined as the dependent variable and fixed and random effects same as above.

The egg hatch rate was analyzed using a generalized binomial linear mixed-effects model fit by maximum likelihood (Laplace approximation) with logit link with the hatch rate defined as dependent variable, cage type, egg origin and their interactions as fixed effects and replicates as a random effect.

A comparison of daily mortality rates between the FAO/IAEA stainless-steel cage and the mass-rearing cage prototype was performed using adult-index data. A constant mortality rate was estimated from plotting the logarithm of counts against time [28]. A Gaussian linear mixed-effects model fit by maximum likelihood was then used to analyze mortality rates, with cage type and square location as fixed effects and replicates as a random effect.

The best model in all analyses was selected based on the lowest corrected Akaike information criterion (AICc), and the significance of fixed effects was tested using the likelihood ratio test [29,30].

All significant differences are based on *p*< 0.05.

## Results

In addition to the type of material used to manufacture the mass-rearing cage prototype, the main difference to the stainless-steel cage was the cleaning system. While in the prototype, containers can be removed and replaced for cleaning, the stainless-steel cage has inlet and outlet valves on the front side. The inlet valve allows the bottom of the cage to be filled with water for egg laying and the outlet valve allows dead pupae and adults to be removed (after adult emergence) and eggs floating in water (remaining in the cage after egg collection) to be collected. The mass-rearing cage prototype weighs about 5 kg compared to 16 kg for the stainless-steel cage and thus the cost of transportation is significantly reduced. Compared to the prototype (~230€ of total cost), the manufacture of the stainless-steel cage only counts for more than 90% of its total cost (2,300€).

**Fig 3:**
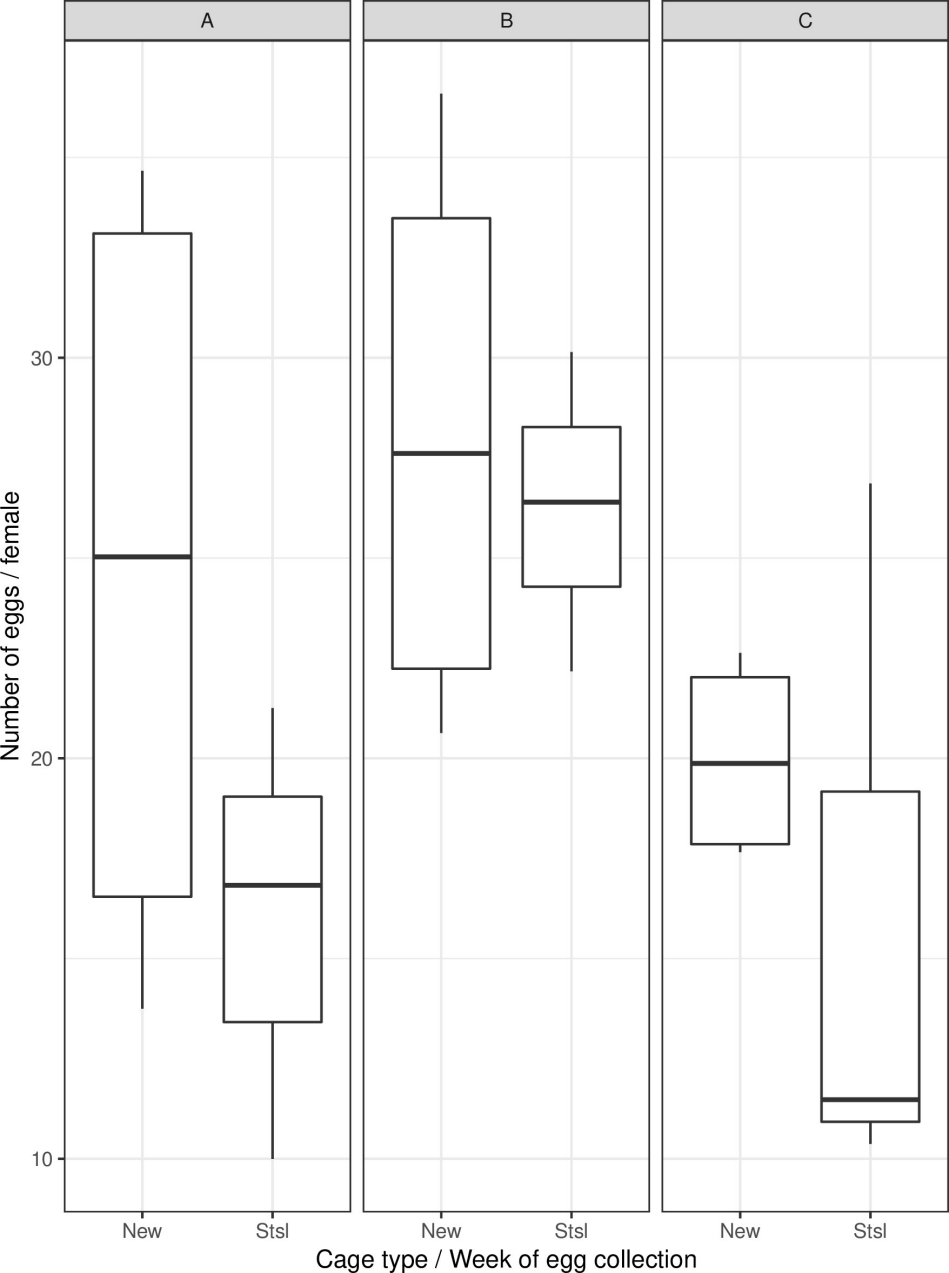
Number of eggs per initial female according to the cage type and week of egg collection. Panels A, B and C indicate weeks 1, 2 and 3 of egg collection respectively. New (n = 12) and Stsl (n = 9) represent the mass-rearing cage prototype and the FAO/IAEA stainless-steel cage respectively and n denotes the number of analyzed samples. The bars represent the median, upper and lower quartiles.

The mass-rearing cage prototype produced a comparable number of eggs per initial female to the FAO/IAEA stainless-steel cage (Table 3, Fig 3, *p* = 0.79). More eggs were collected in both cage types on the second week of egg collection compared to the first and the third weeks (*p* = 0.02).

**Table 3:** Effect of cage type on egg production.

	Value	Std. Error	DF	t-value	*p*
**(Intercept)**	11.5000	2.1455	16	5.3601	0.0001
**Stainless-steel cage**	-0.6886	2.5905	16	-0.2658	0.7938

Std, standard; DF, degrees of freedom

The stainless-steel cage was compared to the mass-rearing cage prototype.

**Fig 4:**
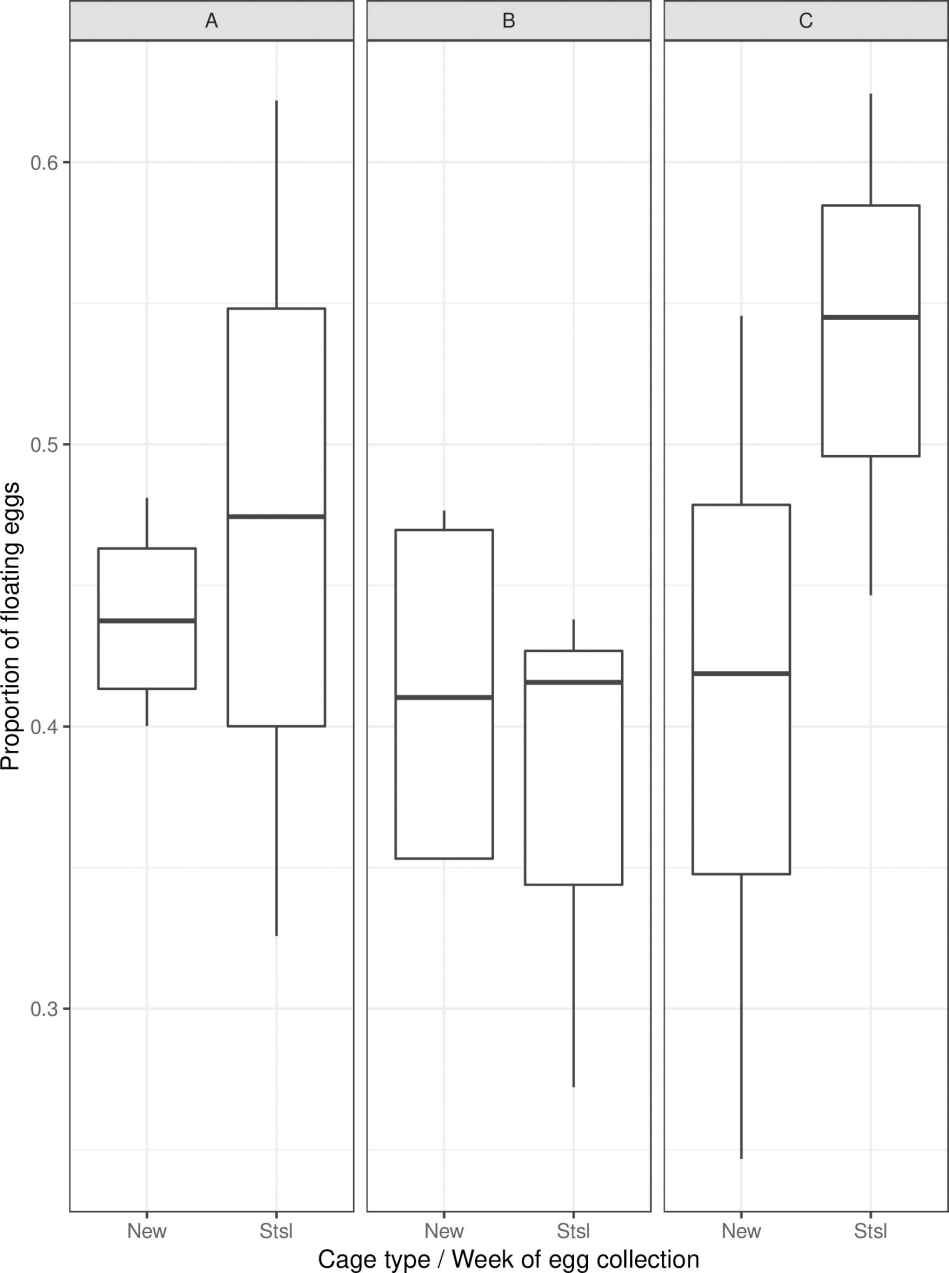
Proportion of floating eggs according to the cage type and week of egg collection. Panels A, B and C indicate weeks 1, 2 and 3 of egg collection respectively. New (n = 12) and Stsl (n = 9) represent cage types, namely the mass-rearing cage prototype and the FAO/IAEA stainless-steel cage, and n denotes the number of analyzed samples. The bars represent the median, upper and lower quartiles.

The mean numbers (± S. E.) of eggs per cage after seven blood feedings/egg batches were 969,789 ± 138,101 and 779,970 ± 123,042, corresponding to 81 ± 11 and 65 ± 10 eggs per female throughout each cage’s active period (32 days), for the prototype and the stainless-steel cage, respectively. However, the proportion of floating eggs in the stainless-steel cage was significantly higher (average 46% of the total egg production) than that of the mass-rearing cage prototype (average 41%) (Table 4, *p*<0.001, Fig 4).

**Table 4:** Proportion of floating eggs in each cage type and week of egg collection.

	Estimate	Std. Error	z value	*p*(>|z|)
**(Intercept)**	-0.3336	0.0579	-5.766	8.14e-09 ***
**Stainless-steel cage**	0.1573	0.0032	49.575	< 2e-16 ***
**week2**	0.0092	0.0024	3.817	0.000135***
**week3**	-0.0087	0.0026	-3.295	0.000984 ***
**Stainless-steel cage: week2**	-0.2776	0.0040	-68.867	< 2e-16 ***
**Stainless-steel cage: week3**	0.1958	0.0044	44.147	< 2e-16 ***

Std, standard

The stainless-steel cage was compared to the mass-rearing cage prototype and the weeks 2 and 3 to the week 1.

The proportion of floating eggs collected from the stainless-steel cage was significantly lower during the second week (Table 4, *p*<0.001) but greater during the third week (Table 4, *p*<0.001) than that of the mass-rearing cage prototype during the first week.

**Fig 5:**
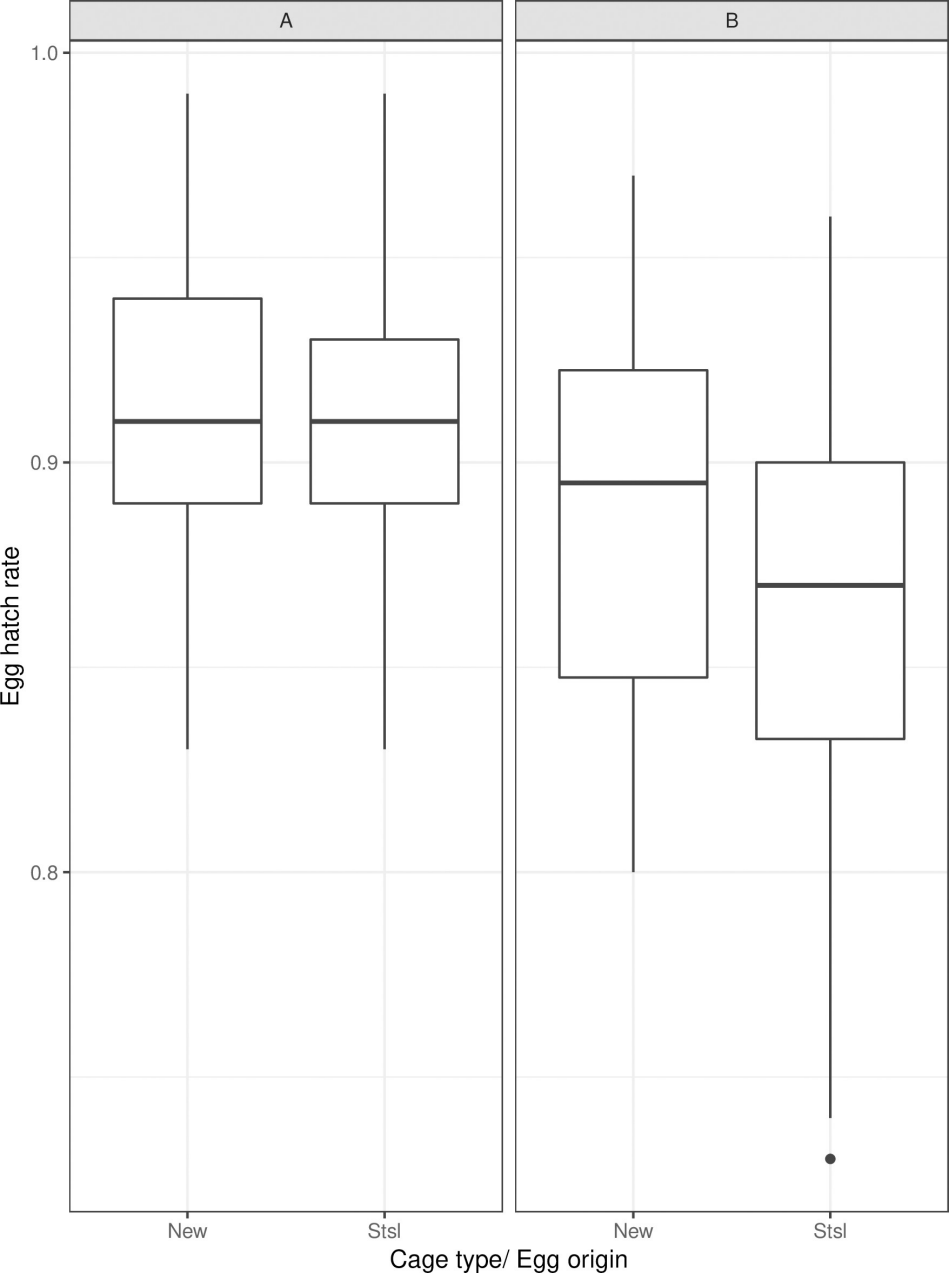
Egg hatch rate according to cage type and egg origin. Panels A and B indicate egg origin (paper and floating respectively). New (paper, n = 72; floating, n = 24) and Stsl (paper, n = 54; floating, n = 18) represent the mass-rearing cage prototype and the FAO/IAEA stainless-steel cage, and n denotes to the number of analyzed samples. The bars represent the median, upper and lower quartiles.

The egg hatch rate was significantly affected by cage type and egg origin. The mass-rearing cage prototype’s egg hatch rate was higher than that of the stainless-steel cage (Table 5, *p* = 0.04, Fig 5).

**Table 5:** Egg hatch rate considering cage type and egg origin.

	Estimate	Std. Error	z value	*p*(>|z|)
**(Intercept)**	2.0067	0.0704	28.505	< 2e-16 ***
**Stainless-steel cage**	-0.1147	0.0560	-2.047	0.0407 *
**paper**	0.3880	0.0566	6.851	7.34e-12 ***

Std, standard

The stainless-steel cage was compared to the mass-rearing cage prototype and eggs collected in seed germination papers to the floating eggs.

The eggs collected on seed germination papers had a higher hatch rate as compared to eggs floating in water (Table 5, *p*<0.001, Fig 5). Egg origin had a greater impact on egg hatch rate compared to cage type.

**Fig 6:**
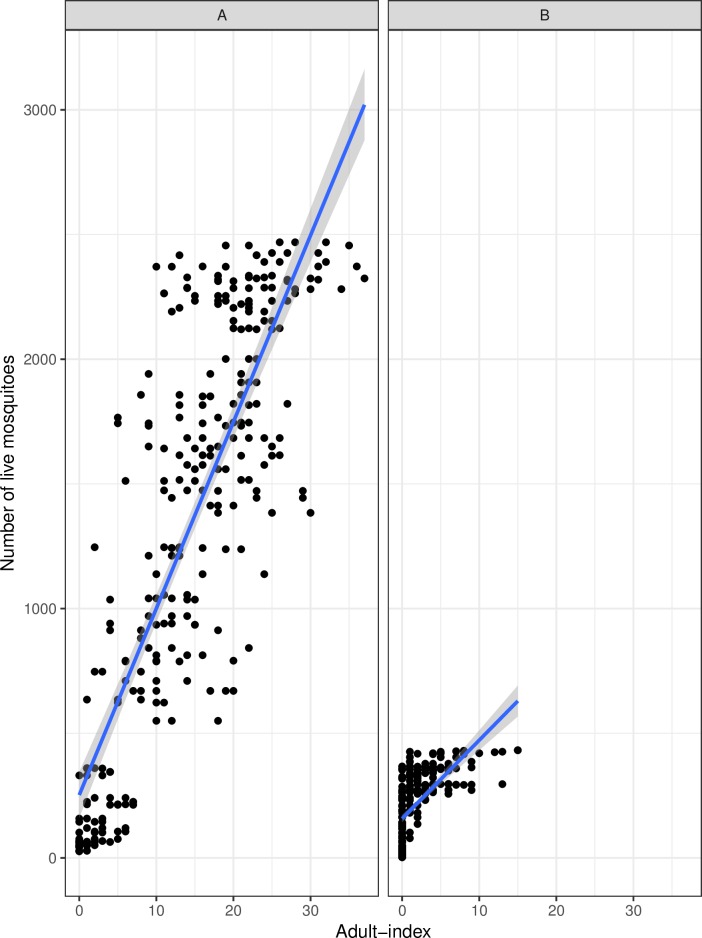
Correlation between daily live female (A) and male (B) mosquitoes and adult-index.

The adult-index was validated and deemed to be a good proxy of mortality. There was a positive correlation between adult-index and daily live mosquitoes in the 30 × 30 × 30 cm cage both for females (Fig 6A, t = 27.53, df = 298, cor = 0.84, *p*<0.0001) and males (Fig 6B, t = 14.67, df = 298, cor = 0.64, *p*<0.0001). Moreover, the best model to predict the mortality from the counts was the one using the mortality estimated from the index only (the effects of the square number and the sex did not improve the model). The predicted mortality was correlated to the observed value (cor = 0.59, t = 3.89, df = 28, *p*<0.0001).

Using this index, no significant difference was observed between the stainless-steel and the mass-rearing prototype cages (Table 6, *p* = 0.53). However, females lived longer than males in both cages (Table 6, *p* = 0.0029). Although the square location impacted the measured mortality (the best model included this factor and its interaction with the cage), no significant effect of the location of the squares was observed (Table 6, *p*˃0.05).

**Table 6:** Survival rates considering sex, cage type and square location.

Parameters	Value	Std. Error	DF	t-value	*p*
**(Intercept)**	0.06461181	0.013413	63	4.816931	0.0000
**Stainless-steel cage**	0.01224230	0.019515	63	0.627343	0.5327
**square2**	-0.00652444	0.018067	63	-0.361126	0.7192
**square3**	-0.03224221	0.018067	63	-1.784599	0.0791
**square4**	0.00721702	0.018067	63	0.399461	0.6909
**square5**	-0.01430899	0.01871	63	-0.764771	0.4473
**square6**	-0.03651868	0.01871	63	-1.95181	0.0554
**male**	0.02534524	0.008177	63	3.099684	0.0029
**Stainless-steel cage: square2**	-0.02032741	0.027598	63	-0.736562	0.4641
**Stainless-steel cage: square3**	0.01997768	0.028387	63	0.703762	0.4842
**Stainless-steel cage: square4**	0.00148962	0.027598	63	0.053976	0.9571
**Stainless-steel cage: square5**	0.00084721	0.028784	63	0.029433	0.9766
**Stainless-steel cage: square6**	0.02191523	0.028784	63	0.761366	0.4493

Std, standard error; DF, degrees of freedom

The stainless-steel cage was compared to the mass-rearing cage prototype; adult male mosquitoes to females; square 1 was as reference compared to the squares 2, 3, 4, 5 and 6.

## Discussion

The ability to colonize and mass rear an insect species in adequate numbers and with relative efficiency are prerequisites for considering an SIT program for that species [31]. We present here a low-cost mass-rearing cage prototype, developed with consideration of the biology of *Ae*. *aegypti*, and to the results of a comparative test with the FAO/IAEA stainless-steel cage, measuring egg production and egg hatch rate. In addition, a new adult-index was validated as a proxy for adult density in a cage, and used to estimate mosquito survival rates. We have shown that the mass-rearing cage prototype is much cheaper (ten times) and as efficient as the FAO/IAEA stainless-steel cage in terms of adult mosquito survival and egg production, and gives a better egg hatch rate. In addition, the mass-rearing cage prototype is easy to handle, clean, and to transport.

In general, increasing the size of adult cages reduces the total time needed to handle a given number of adults and therefore improves cost-time efficiency. For large scale production a large number of cages would be needed, and so the initial investment in a facility would be high, and developing a low-cost cage allows considerable savings. Furthermore, the proposed mass-rearing cage prototype is lighter (~5 kg compared to the 16Kg for the stainless-steel cage) and enables easy handling, installation and cleaning, reducing the workload and number of staff needed. Based on the DRS of 0.8 considered in our study, the mass-rearing cage prototype would hold as many adults as six 30 × 30 × 30 cm standard rearing cages (BugDorm-1 Insect Rearing Cage, Taiwan) which cost €60.54/unit (https://shop.bugdorm.com/insect-rearing-bugdorm-1-insect-cages-c-5_25.html) and would mean routine operations such as blood feeding and egg collection would need to be performed on one cage instead of six. Moreover, no internal handling is required once the cage is fully stocked with pupae, reducing the number of escapees and therefore protecting staff from bites. Although we did not monitor the exact number of escapees, they seemed to be similarly low for both cages. The middle section of the top of the mass-rearing cage prototype also has an opening (180 mm of diameter covered with netting) allowing the use of the Hemotek device (Discovery Workshops, Accrington, UK; [12]), as an alternative to the sausage casings. A study carried out in Brazil has shown that 28 cages of 30 × 30 × 30 cm were needed to produce 4 million eggs per week, [16]. For the same production with two blood feedings per week, only 7 cages of the prototype would be needed assuming that same number of eggs per female would be produced. For a medium scale mass-rearing facility with a production level of 10 million sterile males per week, about 200 cages would be needed. Considering the difference in cost, the mass-rearing cage prototype manufactured locally can reduce initial investments for equipment by more than €350,000 compared to the stainless-steel cage in a facility of this scale. Other materials such as recycled plastic, fiber glass or aluminium could be used as alternatives to further reduce the costs compared to stainless-steel.

Our results did not show any difference in egg production between the cages. However, more eggs were collected during the second week of egg collection. A possible explanation of the delay in egg laying could be female mosquito age, ranging between 3 and 6 days when the first blood feeding occurs. This may lead to differential female blood feeding rates or blood intake volumes, and consequently egg yield, although early studies have reported that both *Ae*. *aegypti* and *Ae*. *albopictus* females take their first bloodmeal from hosts on the second or third day after emergence [32]. In any case, it was found that more females produced eggs in the second week. We recommend reducing pupal loading to one event on the same day to homogenize adult age, blood feeding rates and increase overall blood volume intake, thereby increasing the total egg production in the first two weeks. This would corroborate the results of Zhang et al. [19] who have recommended a two week-cycle for *Ae*. *albopictus* rearing cages in a medium scale rearing facility. Cage structure (height and width) has been shown to play a great role in *Ae*. *albopictus* egg production according to their study. Another advantage of the mass-rearing cage prototype described in this study is that the height can be adjusted according to species, if needed. We plan to test this cage for mass-rearing *Ae*. *albopictus* in the near future and to adapt its structure to accommodate the mass-rearing of *Anopheles arabiensis*, particularly by adapting the oviposition containers.

Usually *Aedes* females deposit their eggs above the water level in mainly dark-coloured, man-made containers in and around cities [33,34], and so in rearing facilities eggs are collected on a moist substrate. In our study, the proportion of eggs collected from the water rather than on the wet paper provided found to be above 40% for both prototype and stainless-steel cages, though a greater proportion of floating eggs was found in the stainless-steel cage. Similar results have been observed in *Ae*. *aegypti* in the laboratory and in a semi-field setting where a high percentage of floating eggs was observed in the larval sites [35]. Another study described that in field traps, *Ae*. *aegypti* females oviposited 22.7% of their eggs on the water surface [36]. The presence of floating eggs may be due to the phenomenon called skip oviposition behaviour [37], and that the limited number of oviposition papers per cage might play a role in the females’ choice of laying eggs on the water or the paper substrate. It has been shown that gravid female *Ae*. *aegypti* favour larval sites that already contain eggs or larvae [38]. Given that the stainless-steel cage has a continuous presence of larvae in the water, the gravid females may lay a larger proportion of their eggs on the water surface instead of the provided paper substrate compared to females in the mass-rearing cage prototype, where no larvae are present in the water.

The mean egg hatch rate for both cages was above 85%, with eggs collected from the mass-rearing prototype cages showing a better overall hatch rate after egg storage for two weeks after oviposition, contradicting an observation made by Zhang et al. [19] who observed that the cage structure had no impact on egg hatch rate in their *Ae*. *albopictus* strain. However, in our study, egg hatch rate was compared between the eggs found on oviposition papers and floating eggs. The latter showed a decrease in hatch rate when they were collected from the stainless-steel cage. This difference might be due to the pre-existing eggs and larvae remaining from the first egg collection in the stainless-steel cage. Huang et al. [39] observed that older (third and fourth instar) *An*. *gambiae* larvae readily eat *An*. *gambiae* eggs and first instar larvae. The quality of the water, and egg collection and storage methods affect the egg maturation process and therefore hatch rate.

Another major disadvantage of the stainless-steel cage is that the floating eggs are difficult to recover. Although more than 4 L of water was used to rinse the bottom of the cage, a significant number of eggs remained in the cage and hatched prematurely. This causes a loss in yield that is uneconomical in a mass-production factory. Gomes et al. [40] conducted a laboratory experiment and indicated that eggs deposited on the water surface showed faster hatching which could favour faster establishment in wild habitats. Dead mosquitoes (adults, pupae that failed to emerge, and larvae resulting from premature hatching) in the stainless-steel cage could lead to bacterial growth resulting in a rapid decrease in oxygen levels in the water, stimulating egg hatch [41]. The mass-rearing cage prototype has the advantage that egg containers are removed and replaced so that no unwanted egg hatch occurs.

Monitoring production and quality of mass-reared insects is important in any rearing facility. Although attention is given to the larval rearing stage to ensure the quality of resulting adults, it is also important to have procedures to monitor and follow the size of the egg producing colony. Miller and Weidhaas [42] suggested that the adult survival rate is the most important factor determining the stability of the population and total egg production. The adult-index method described and validated here could be used as a simple tool to monitor the survival of a cage population as an indicator for the overall status of the mass-rearing colony. A daily adult-index check of one mass-rearing cage would take less than 5 minutes depending on when in the rearing cycle it was. Provided that the index is calibrated for each given environment and strain, any unusual increase in mortality may be spotted based on a decrease in the daily recorded adult index. Other methods based on geometric morphometric analysis (wing size and shape) have been used as a criterion to the quality of mass-rearing procedures [43]. Although the prototype cage structure is different to that of the FAO/IAEA stainless-steel cage, male and female mosquitoes survived equally well in either cage. This could be due to the density of caged mosquitoes being equal and optimized for longevity [8,15]. A DRS value of 0.9 has been used for the mass-production of *Ae*. *aegypti* in Brazil [16] whereas a DRS value of 0.8 that was measured in our study. Male mosquitoes showed higher mortality rates compared to females, which is likely to be, among other factors, due to mating-related stress. It is well known that mating is costly in terms of energy [44,45] and a recent study pointed out that an increase in female survival could be due to male-derived seminal fluid molecules transferred to the female [46].

## Conclusions

We have shown in this study that the proposed mass-rearing cage prototype has several advantages in comparison to the previous stainless-steel cage developed by the FAO/IAEA and that the adult-index is a quick and reliable proxy of mosquito survival rates which could be used in mass-rearing settings. Although an improved mass-rearing cage has been developed, further studies on blood feeding optimization are needed. In addition, egg collection can be optimized by increasing the number of egg papers to reduce the number of floating eggs. Moving egg collection containers to the side instead of along the bottom of the cage will also improve the design and further minimize escapees as well as work load. To better fit the cage mesh to the cage structure, another prototype with Hook-and-loop fasteners (VELCRO) is under investigation and will soon be tested. Alternative materials to lower the production cost of the new mass-rearing cage prototype could also be investigated for developing countries. The mass-rearing cage prototype design and drawings are available for open access on the IAEA website and may be used to locally produce the cages for use in SIT programmes or indeed large-scale rearing for any purpose.

## Supporting information

S1 Fig The overall view of the mass-rearing cage prototype complete structure drawings. (PDF)

S2 Fig The assembly of the mass-rearing cage prototype complete structure. (PDF)

S3 Fig The overall view of the upper plate of the mass-rearing cage prototype. (PDF)

S4 Fig The assembly of the upper plate of the mass-rearing cage prototype. (PDF)

S5 Fig Drawings and dimensions of the upper plate-top part. (PDF)

S6 Fig Drawings and dimensions of the upper plate-middle part. (PDF)

S7 Fig Drawings and dimensions of the upper plate-bottom part. (PDF)

S8 Fig Drawings and dimensions of the upper plate- reinforcement part. (PDF)

S9 Fig The overall view of the bottom plate of the mass-rearing cage prototype. (PDF)

S10 Fig The assembly of the bottom plate of the mass-rearing cage prototype. (PDF)

S11 Fig Drawings and dimensions of the upper plate-top and bottom parts. (PDF)

S12 Fig Drawings and dimensions of the bottom plate-middle part. (PDF)

S13 Fig Drawings and dimensions of the bottom plate—Narrow guidance part. (PDF)

S14 Fig Drawings and dimensions of the bottom plate—Wide guidance part. (PDF)

S15 Fig Drawings and dimensions of egg collection container—Overall view. (PDF)
