# Supplementary material for: Reducing the cost and assessing the performance of a novel adult mass-rearing cage for the dengue, chikungunya, yellow fever and Zika vector, Aedes aegypti (Linnaeus)
Source: PLoS Negl Trop Dis. 2019 Sep 25;13(9):e0007775. doi: 10.1371/journal.pntd.0007775 (PMC6779276; doi:10.1371/journal.pntd.0007775)
Supplement: S1 Fig — (PDF) [file pntd.0007775.s001.pdf]

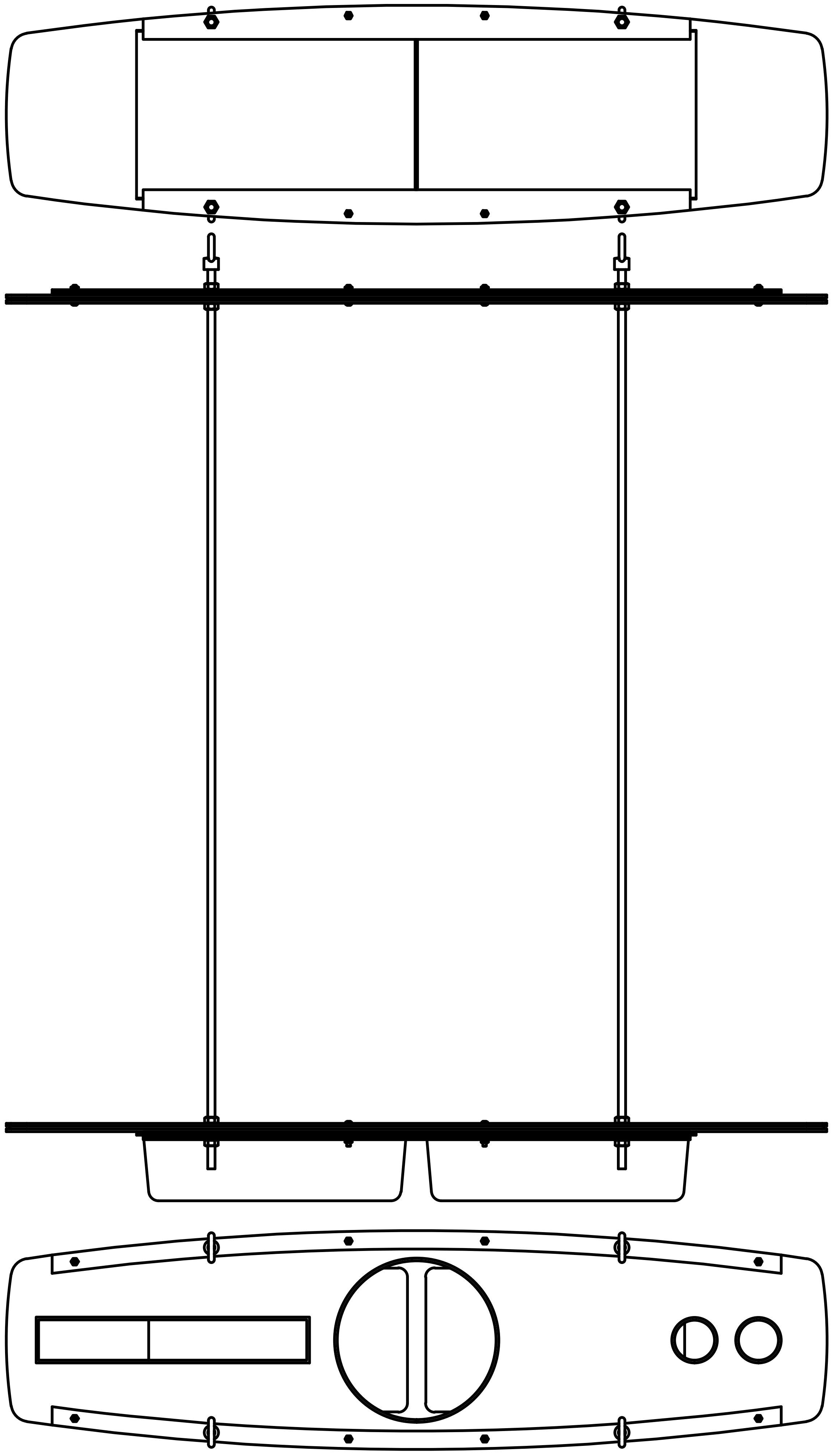

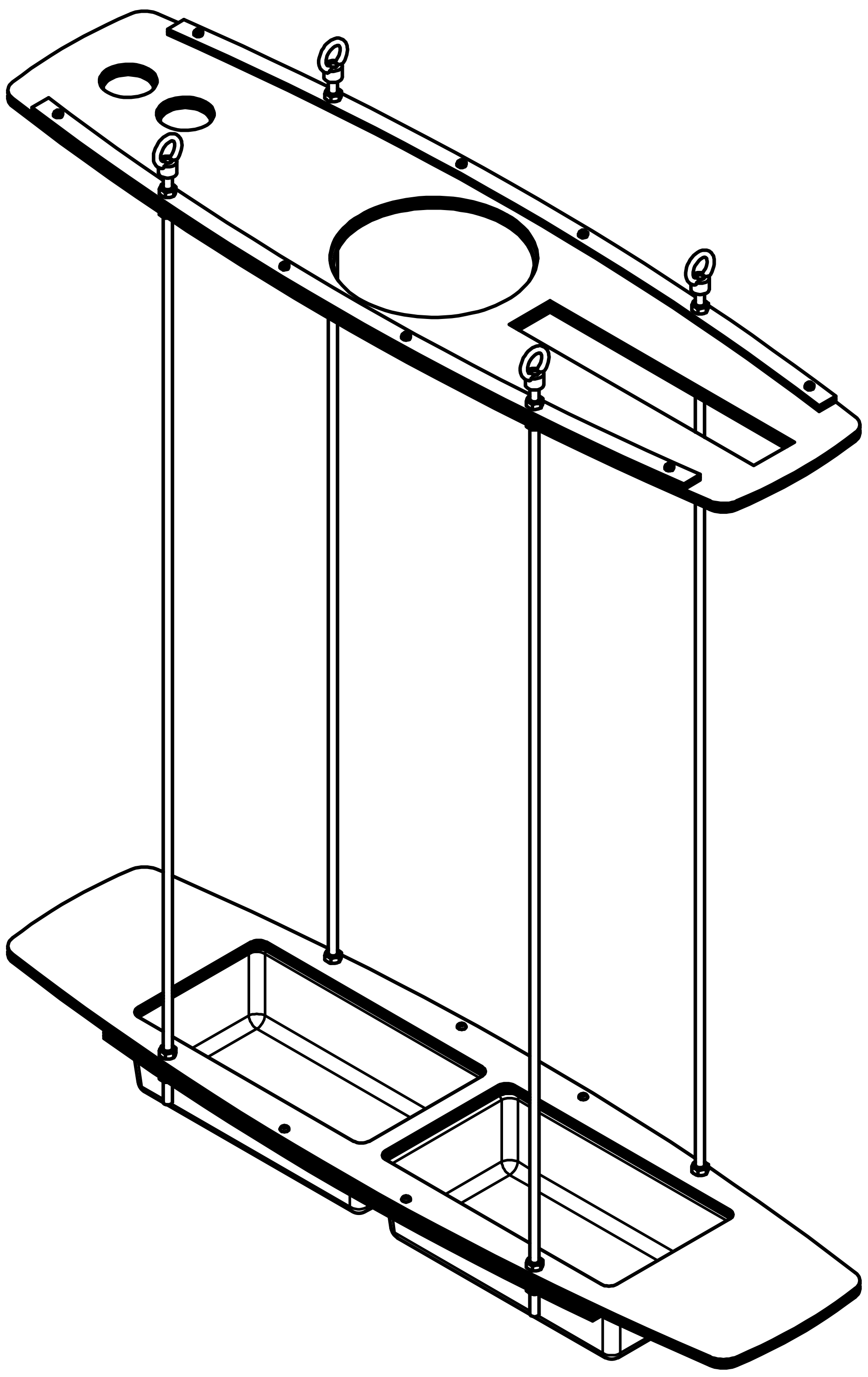

	Name	Date		
Designed	G. Salvador-Herranz	10/12/2018		Insect Pest Control Section
Revised	R. Argilés	10/12/2018		
Scale 1:6 mm	PMMA Aedes Cage v1			Number AEDES_CAGE_V1
	Complete Structure - Overall View			Sheet 1/15
