# Supplementary material for: Reducing the cost and assessing the performance of a novel adult mass-rearing cage for the dengue, chikungunya, yellow fever and Zika vector, Aedes aegypti (Linnaeus)
Source: PLoS Negl Trop Dis. 2019 Sep 25;13(9):e0007775. doi: 10.1371/journal.pntd.0007775 (PMC6779276; doi:10.1371/journal.pntd.0007775)
Supplement: S2 Fig — (PDF) [file pntd.0007775.s002.pdf]

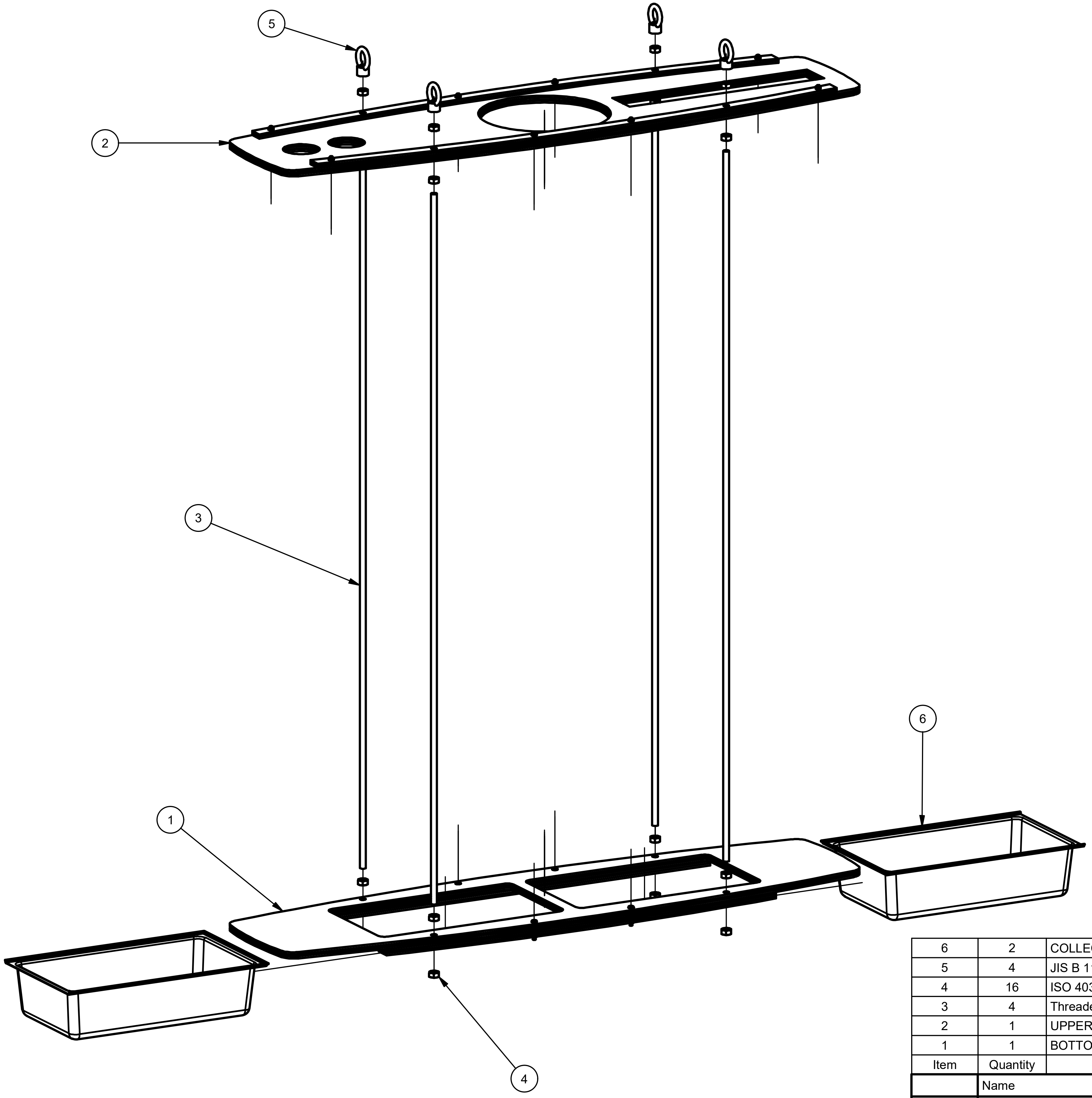

Item	Quantity	Part	Description
6	2	COLLECT_TRAY	Egg collection tray. ABS 1mm.
5	4	JIS B 1169 - M 8	Eyebolt nut M8
4	16	ISO 4032 - M8	Hexagonal nut M8. Stainless steel.
3	4	Threaded rods GB/T 15389-1994 M8 x 1000	Threaded rods M8. Stainless steel.
2	1	UPPER_PLATE	PMMA Upper Plate
1	1	BOTTOM_PLATE	PMMA Bottom Plate

	Name	Date
Designed	G. Salvador-Herranz	10/12/2018
Revised	R. Argilés	10/12/2018

Joint FAO/IAEA Programme

Insect Pest Control Section

Scale: 1:6 mm

PMMA Aedes Cage v1

Complete Structure - Assembly

Number: AEDES_CAGE_V1

Sheet: 2/15
