# Supplementary material for: Reducing the cost and assessing the performance of a novel adult mass-rearing cage for the dengue, chikungunya, yellow fever and Zika vector, Aedes aegypti (Linnaeus)
Source: PLoS Negl Trop Dis. 2019 Sep 25;13(9):e0007775. doi: 10.1371/journal.pntd.0007775 (PMC6779276; doi:10.1371/journal.pntd.0007775)
Supplement: S4 Fig — (PDF) [file pntd.0007775.s004.pdf]

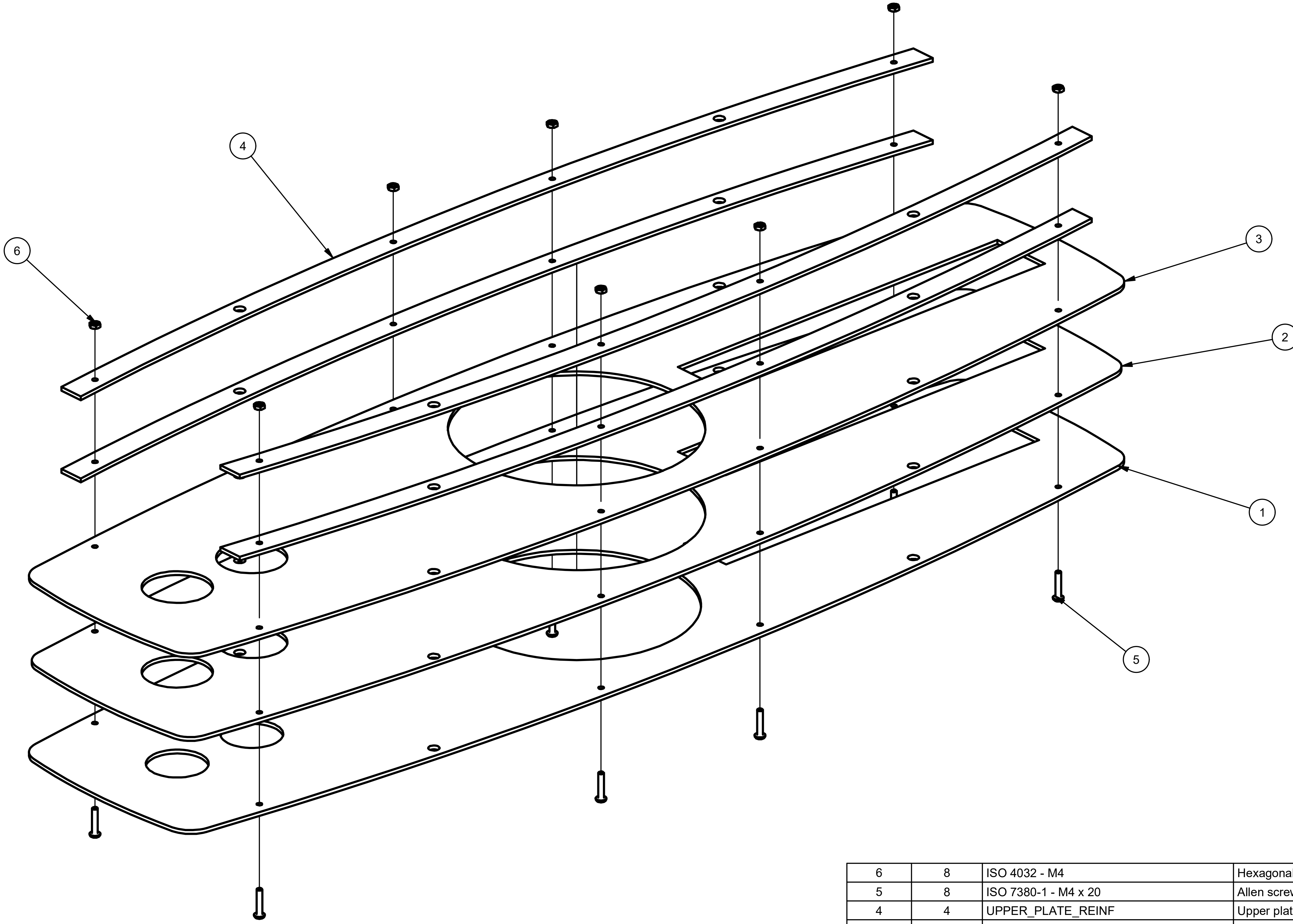

Item	Quantity	Part	Description
6	8	ISO 4032 - M4	Hexagonal nut M4, Stainless steel.
5	8	ISO 7380-1 - M4 x 20	Allen screw M4x20mm. Stainless steel.
4	4	UPPER_PLATE_REINF	Upper plate, reinforcement part. PMMA 3mm.
3	1	UPPER_PLATE_3	Upper plate, top part. PMMA 3mm.
2	1	UPPER_PLATE_2	Upper plate, middle part. PMMA 3mm.
1	1	UPPER_PLATE_1	Upper plate, bottom part. PMMA 3mm.

	Name	Date
Designed	G. Salvador-Herranz	10/12/2018
Revised	R. Argilés	10/12/2018

Joint FAO/IAEA Programme Nuclear Techniques in Food and Agriculture

Insect Pest Control Section

Scale 1:3 mm

PMMA Aedes Cage v1

Upper Plate - Assembly

Number AEDES_CAGE_V1

Sheet 4/15
