# Supplementary material for: Reducing the cost and assessing the performance of a novel adult mass-rearing cage for the dengue, chikungunya, yellow fever and Zika vector, Aedes aegypti (Linnaeus)
Source: PLoS Negl Trop Dis. 2019 Sep 25;13(9):e0007775. doi: 10.1371/journal.pntd.0007775 (PMC6779276; doi:10.1371/journal.pntd.0007775)
Supplement: S6 Fig — (PDF) [file pntd.0007775.s006.pdf]

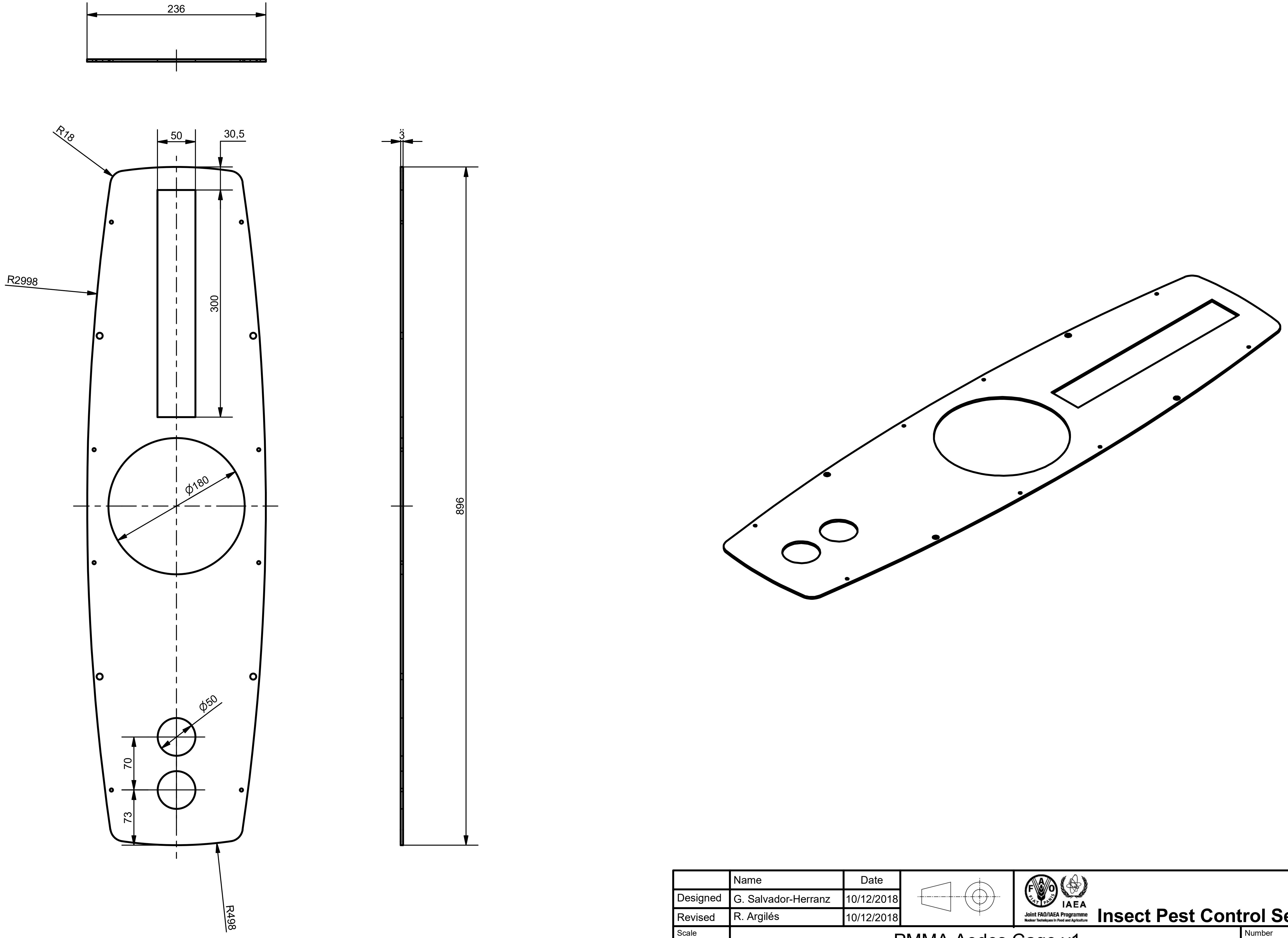

	Name	Date
Designed	G. Salvador-Herranz	10/12/2018
Revised	R. Argilés	10/12/2018

Joint FAO/IAEA Programme

Insect Pest Control Section

Scale: 1:5 mm

PMMA Aedes Cage v1

Upper Plate - Middle Part (UPPER_PLATE_2)

Number: AEDES_CAGE_V1

Sheet: 6/15
