# Supplementary material for: Reducing the cost and assessing the performance of a novel adult mass-rearing cage for the dengue, chikungunya, yellow fever and Zika vector, Aedes aegypti (Linnaeus)
Source: PLoS Negl Trop Dis. 2019 Sep 25;13(9):e0007775. doi: 10.1371/journal.pntd.0007775 (PMC6779276; doi:10.1371/journal.pntd.0007775)
Supplement: S7 Fig — (PDF) [file pntd.0007775.s007.pdf]

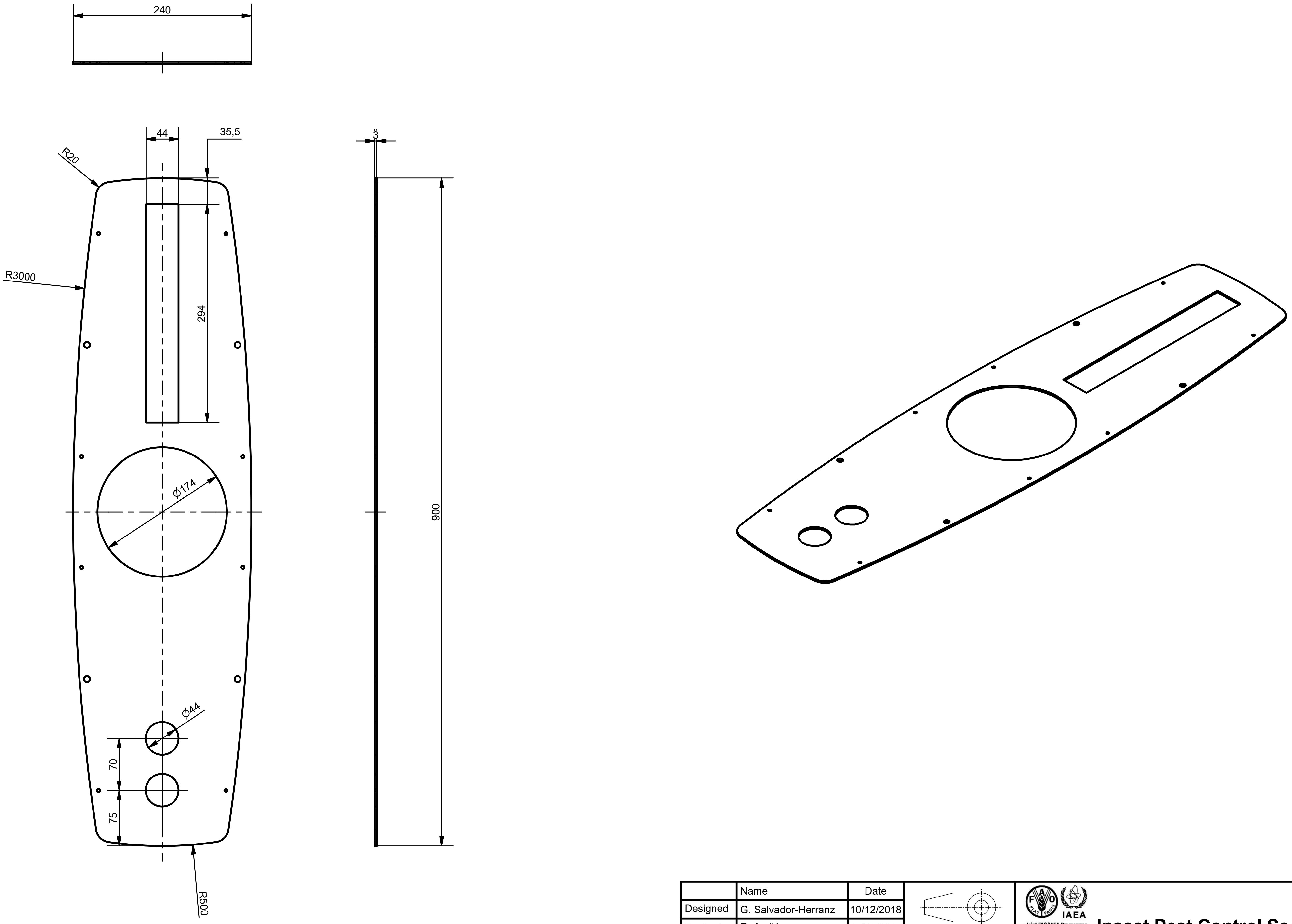

	Name	Date
Designed	G. Salvador-Herranz	10/12/2018
Revised	R. Argilés	10/12/2018

Insect Pest Control Section

Joint FAO/IAEA Programme

Scale		Number
1:5 mm	PMMA Aedes Cage v1	AEDES_CAGE_V1
	Upper Plate - Bottom Part (UPPER_PLATE_1)	Sheet 7/15
