# Supplementary material for: Reducing the cost and assessing the performance of a novel adult mass-rearing cage for the dengue, chikungunya, yellow fever and Zika vector, Aedes aegypti (Linnaeus)
Source: PLoS Negl Trop Dis. 2019 Sep 25;13(9):e0007775. doi: 10.1371/journal.pntd.0007775 (PMC6779276; doi:10.1371/journal.pntd.0007775)
Supplement: S8 Fig — (PDF) [file pntd.0007775.s008.pdf]

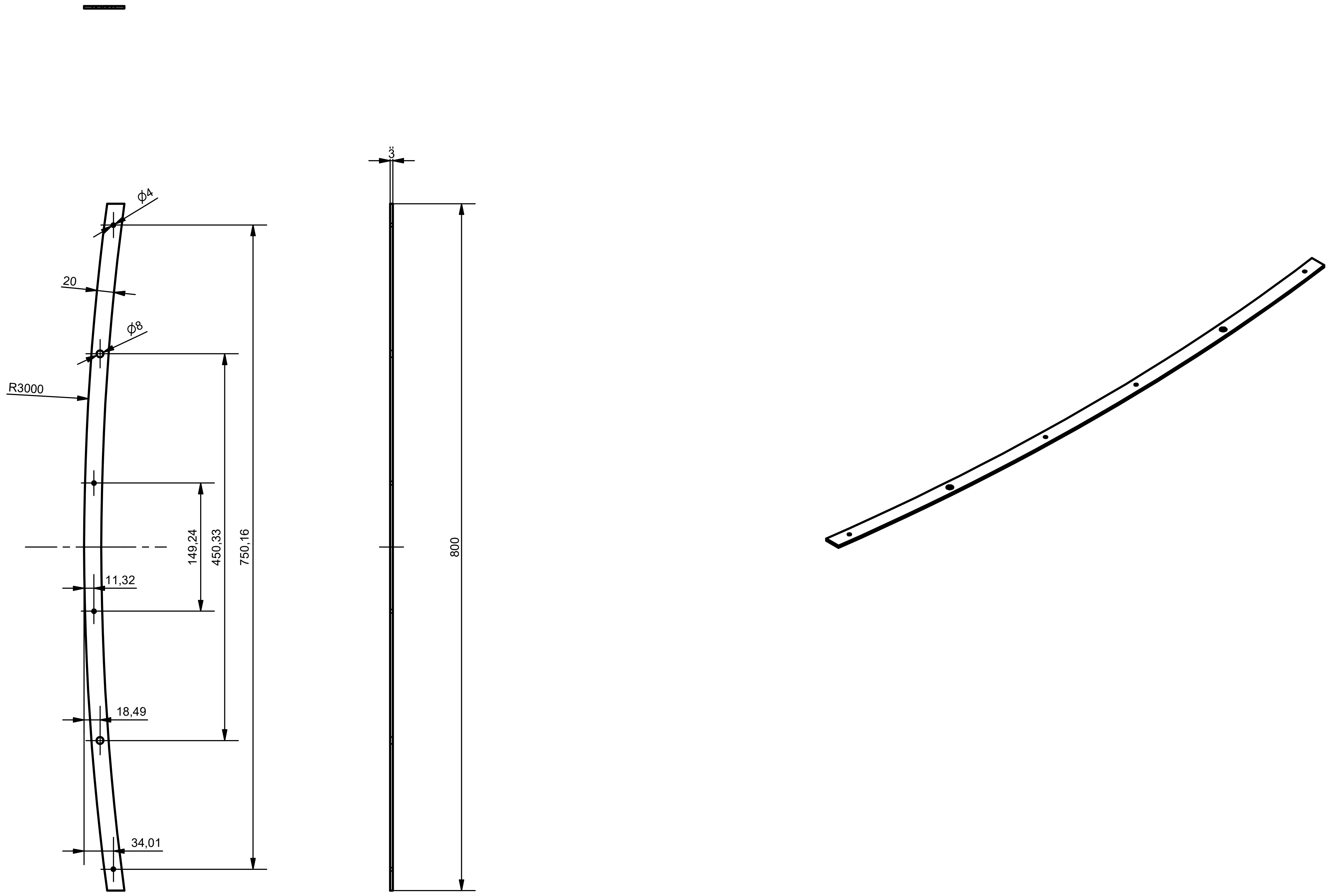


| | Name | Date | | |
|---|---|---|---|---|
| Designed | G. Salvador-Herranz | 10/12/2018 | | Insect Pest Control Section |
| Revised | R. Argilés | 10/12/2018 | | |
| Scale 1:5 mm | PMMA Aedes Cage v1 | | | Number AEDES_CAGE_V1 |
| | Upper Plate - Reinforcement Part (UPPER_PLATE_REINF) | | | Sheet 8/15 |
