# Supplementary material for: Reducing the cost and assessing the performance of a novel adult mass-rearing cage for the dengue, chikungunya, yellow fever and Zika vector, Aedes aegypti (Linnaeus)
Source: PLoS Negl Trop Dis. 2019 Sep 25;13(9):e0007775. doi: 10.1371/journal.pntd.0007775 (PMC6779276; doi:10.1371/journal.pntd.0007775)
Supplement: S10 Fig — (PDF) [file pntd.0007775.s010.pdf]

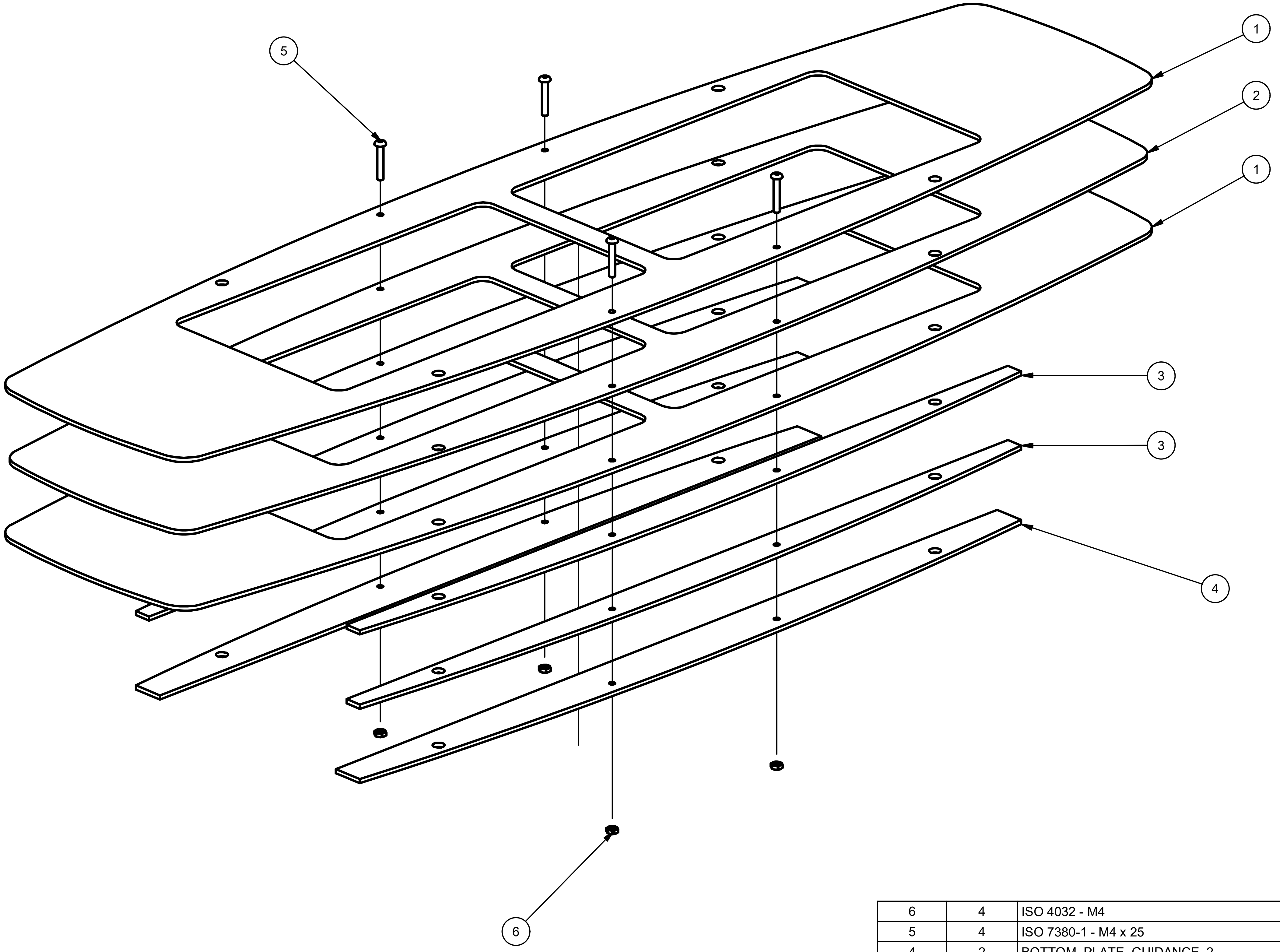

Item	Quantity	Part	Description
6	4	ISO 4032 - M4	Hexagonal Nut M4. Sainless steel.
5	4	ISO 7380-1 - M4 x 25	Allen screw M4x25mm. Stainless steel.
4	2	BOTTOM_PLATE_GUIDANCE_2	Wide guidance part. PMMA 3mm.
3	4	BOTTOM_PLATE_GUIDANCE_1	Narrow guidance part. PMMA 3mm.
2	1	BOTTOM_PLATE_2	Middle plate. PMMA 3mm
1	2	BOTTOM_PLATE_1	Top and bottom plates. PMMA 3mm

	Name	Date
Designed	G. Salvador-Herranz	10/12/2018
Revised	R. Argilés	10/12/2018

Joint FAO/IAEA Programme

Insect Pest Control Section

Scale 1:3 mm

PMMA Aedes Cage v1

Bottom Plate - Assembly

Number AEDES_CAGE_V1

Sheet 10/15
