# Supplementary material for: Reducing the cost and assessing the performance of a novel adult mass-rearing cage for the dengue, chikungunya, yellow fever and Zika vector, Aedes aegypti (Linnaeus)
Source: PLoS Negl Trop Dis. 2019 Sep 25;13(9):e0007775. doi: 10.1371/journal.pntd.0007775 (PMC6779276; doi:10.1371/journal.pntd.0007775)
Supplement: S11 Fig — (PDF) [file pntd.0007775.s011.pdf]

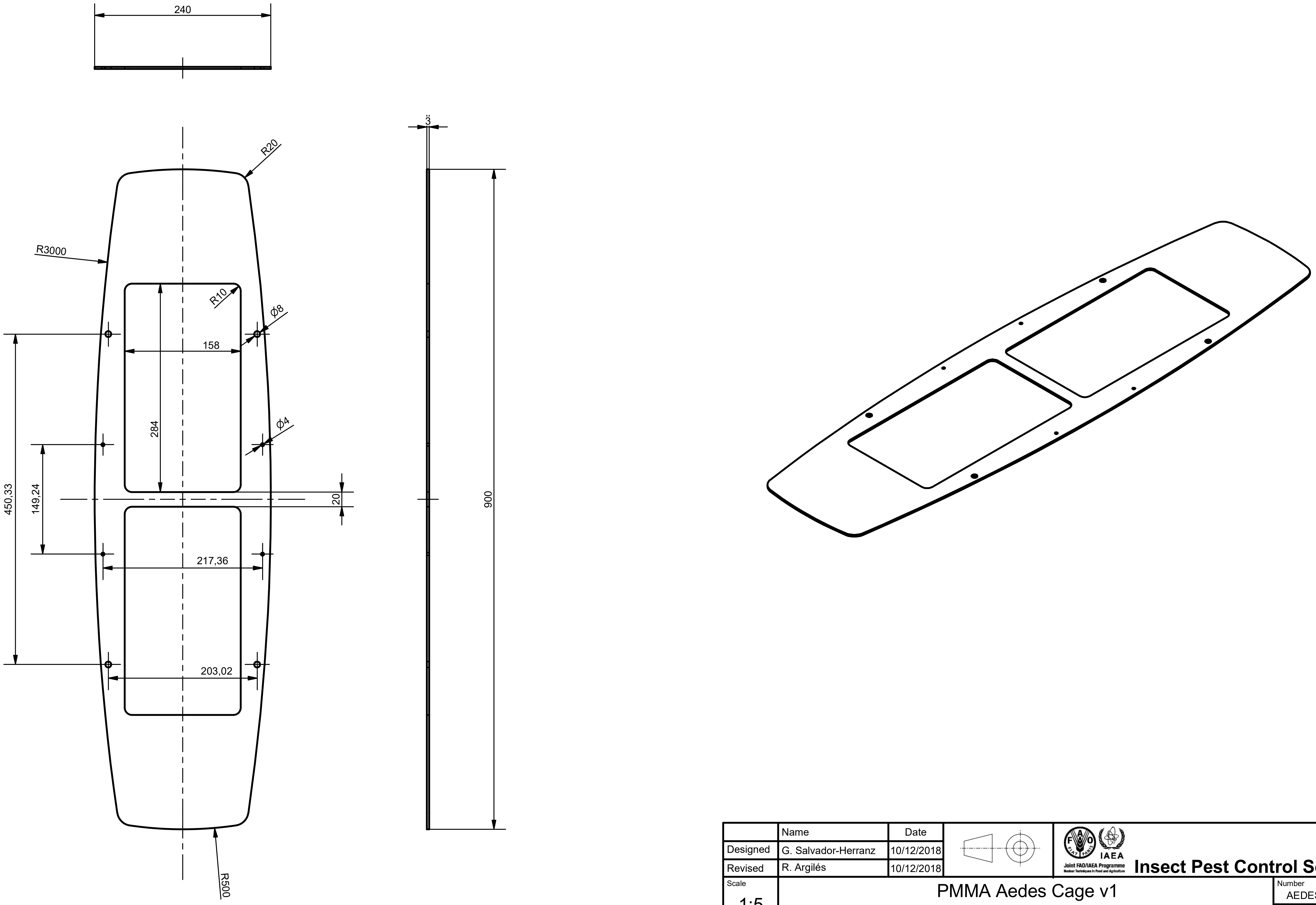

	Name	Date			
Designed	G. Salvador-Herranz	10/12/2018		Joint FAO/IAEA Programme	Insect Pest Control Section
Revised	R. Argilés	10/12/2018			
Scale 1:5 mm	PMMA Aedes Cage v1<br>Bottom Plate - Top and Bottom Parts (BOTTOM_PLATE_1)				Number AEDES_CAGE_V1<br>Sheet 11/15
