# Supplementary material for: Reducing the cost and assessing the performance of a novel adult mass-rearing cage for the dengue, chikungunya, yellow fever and Zika vector, Aedes aegypti (Linnaeus)
Source: PLoS Negl Trop Dis. 2019 Sep 25;13(9):e0007775. doi: 10.1371/journal.pntd.0007775 (PMC6779276; doi:10.1371/journal.pntd.0007775)
Supplement: S13 Fig — (PDF) [file pntd.0007775.s013.pdf]

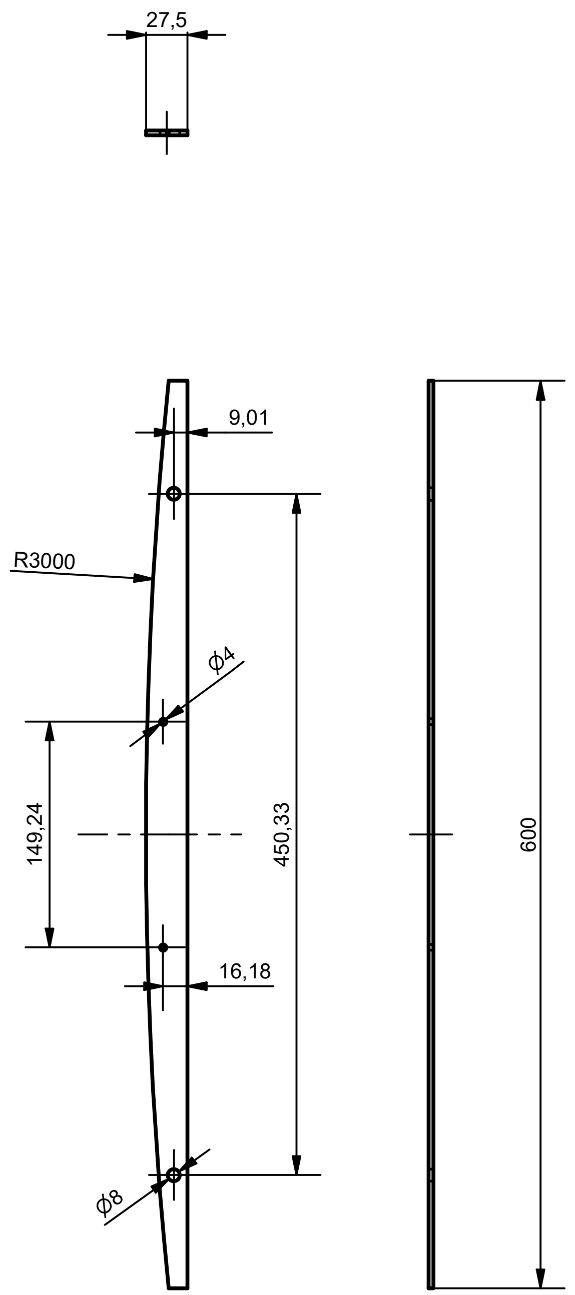

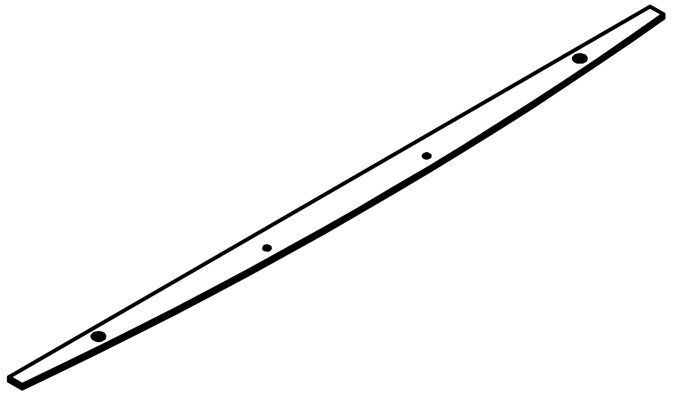

	Name	Date
Designed	G. Salvador-Herranz	10/12/2018
Revised	R. Argilés	10/12/2018

Joint FAO/IAEA Programme

Insect Pest Control Section

Scale	Title	Number / Sheet
1:5 mm	PMMA Aedes Cage v1	Number: AEDES_CAGE_V1
	Bottom Plate - Narrow Guidance Part (BOTTOM_PLATE_GUIDANCE_1)	Sheet: 13/15
