# Supplementary material for: Reducing the cost and assessing the performance of a novel adult mass-rearing cage for the dengue, chikungunya, yellow fever and Zika vector, Aedes aegypti (Linnaeus)
Source: PLoS Negl Trop Dis. 2019 Sep 25;13(9):e0007775. doi: 10.1371/journal.pntd.0007775 (PMC6779276; doi:10.1371/journal.pntd.0007775)
Supplement: S14 Fig — (PDF) [file pntd.0007775.s014.pdf]

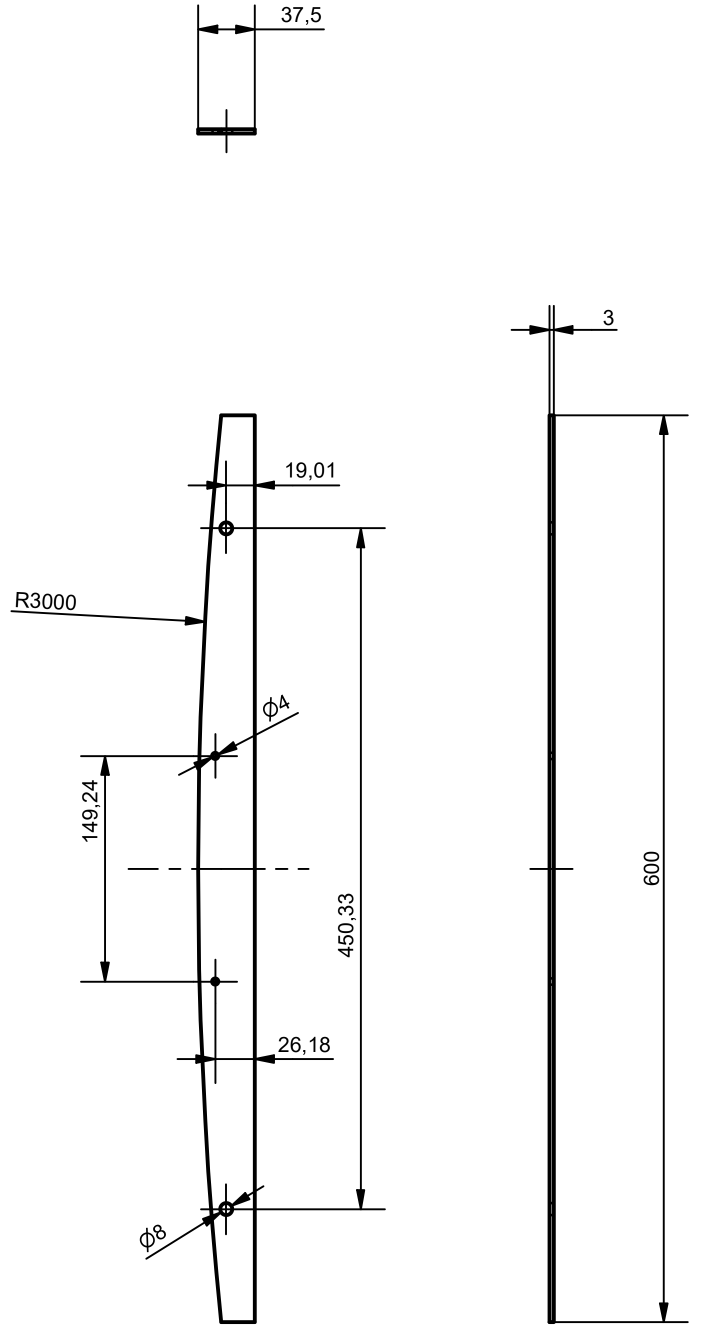

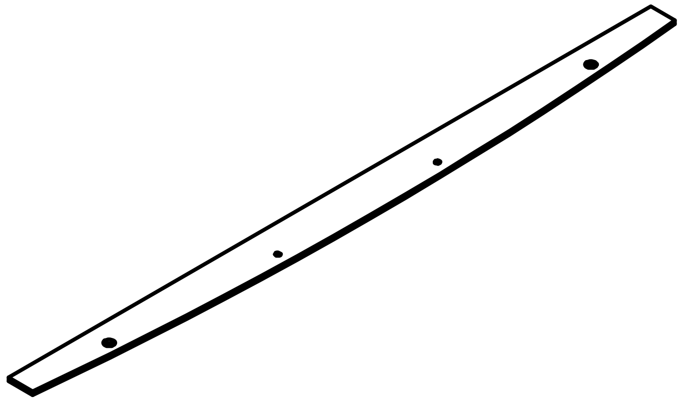

	Name	Date		
Designed	G. Salvador-Herranz	10/12/2018		Joint FAO/IAEA Programme
Revised	R. Argilés	10/12/2018		**Insect Pest Control Section**
Scale 1:5 mm	PMMA Aedes Cage v1<br>Bottom Plate - Wide Guidance Part (BOTTOM_PLATE_GUIDANCE_2)			Number AEDES_CAGE_V1<br>Sheet 14/15
